# Uncovering the therapeutic potential of green pea waste in breast cancer: a multi-target approach utilizing LC-MS/MS metabolomics, molecular networking, and network pharmacology

**DOI:** 10.1186/s12906-024-04669-x

**Published:** 2024-10-31

**Authors:** Asmaa M. Khalil, Omar M. Sabry, Hesham I. El-Askary, Soheir M. El Zalabani, Basma M. Eltanany, Laura Pont, Fernando Benavente, Ahmed F. Mohamed, Nesrin M. Fayek

**Affiliations:** 1 Department of Pharmacognosy, Faculty of Pharmacy, Cairo University, Cairo, 11562 Egypt; 2 Department of Pharmacognosy, Faculty of Pharmacy, Heliopolis University, Cairo, 4645241 Egypt; 3 Department of Pharmaceutical Analytical Chemistry, Faculty of Pharmacy, Cairo University, Cairo, 11562 Egypt; 4 Department of Chemical Engineering and Analytical Chemistry, Institute for Research on Nutrition and Food Safety (INSA·UB), University of Barcelona, Barcelona, 08028 Spain; 5 Serra Húnter Program, Generalitat de Catalunya, Barcelona, 08007 Spain; 6 Department of Pharmacology and Toxicology, Faculty of Pharmacy, Cairo University, Cairo, 11562 Egypt; 7 Faculty of Pharmacy, King Salman International University (KSIU), Ras Sedr, 46612 Egypt

**Keywords:** Breast cancer, Green pea, LC-MS/MS metabolomics, Molecular networking, Network pharmacology, Waste valorization

## Abstract

**Background Pisum sativum:**

(PS) is a universal legume plant utilized for both human and animal consumption, particularly its seeds, known as green peas. The processing of PS in food industries and households produces a significant amount of waste that needs to be valorized.

**Methods:**

In this study, the metabolite profiles of the 70% ethanolic extracts of PS wastes, namely peels (PSP) and a combination of leaves and stems (PSLS), were investigated by liquid chromatography-electrospray ionization-quadrupole time-of-flight tandem mass spectrometry (LC-ESI-QTOF-MS/MS) followed by molecular networking.

**Results:**

Different classes of metabolites were identified, being flavonoids and their derivatives, along with phenolic acids, the most abundant categories. Additionally, a comprehensive network pharmacology strategy was applied to elucidate potentially active metabolites, key targets, and the pathways involved in cytotoxic activity against breast cancer. This cytotoxic activity was investigated in MCF-7 and MCF-10a cell lines. Results revealed that PSLS extract exhibited a potent cytotoxic activity with a good selectivity index (IC_50 =_ 17.67 and selectivity index of 3.51), compared to the reference drug doxorubicin (IC_50 =_ 2.69 µg/mL and selectivity index of 5.28). Whereas PSP extract appeared to be less potent and selective (IC_50 =_ 32.92 µg/mL and selectivity index of 1.62). A similar performance was also observed for several polyphenolics isolated from the PSLS extract, including methyl cis *p-*coumarate, trans *p-*coumaric acid, and liquiritigenin/ 7-methyl liquiritigenin mixture. Methyl cis *p-*coumarate showed the most potent cytotoxic activity against MCF-7 cell line and the highest selectivity (IC_50 =_ 1.18 µg/mL (6.91 µM) and selectivity index of 27.42). The network pharmacology study revealed that the isolated compounds could interact with several breast cancer-associated protein targets including carbonic anhydrases 1, 2, 4, 9, and 12, as well as aldo-keto reductase family 1 member B1, adenosine A3 receptor, protein tyrosine phosphatase non-receptor type 1, and estrogen receptor 2.

**Conclusion:**

The uncovered therapeutic potential of PSLS and its metabolite constituents pave the way for an efficient and mindful PS waste valorization, calling for further in-vitro and in-vivo research.

**Graphical Abstract:**

**Figure:**
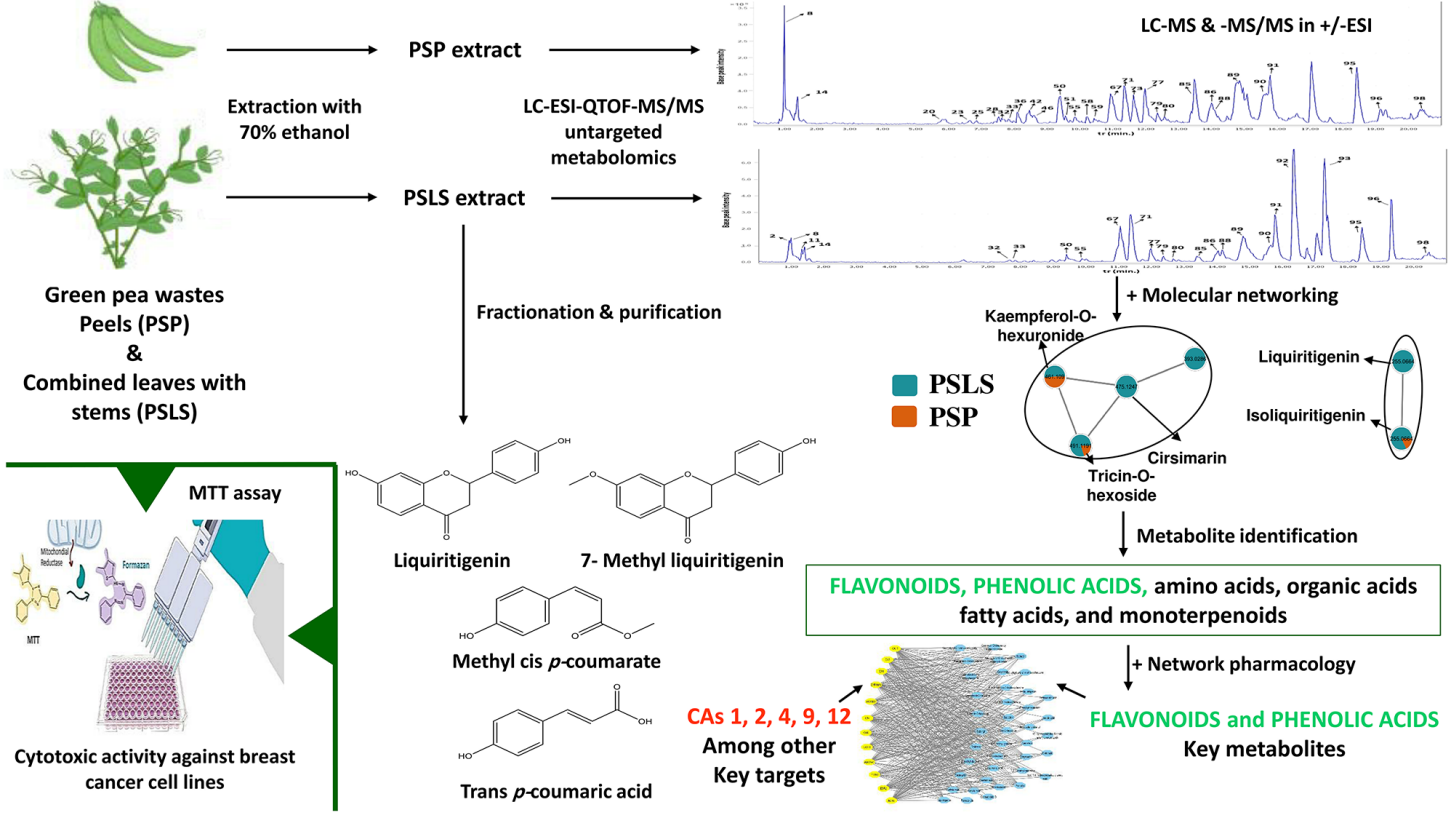

**Supplementary Information:**

The online version contains supplementary material available at 10.1186/s12906-024-04669-x.

## Background

*Pisum sativum* (PS) is a well-known legume plant cultivated worldwide as a source of food for both humans and animals. With its remarkable adaptability, PS has a vast geographical distribution, making it a staple crop in many regions of the world [1]. Its small, spherical seeds, commonly known as green peas, are valued as low-cost source of several nutrients, including vitamins, minerals, proteins, and complex carbohydrates [2]. Moreover, PS seeds have been used traditionally for various medicinal purposes, such as treating acne, diabetes, hemorrhoids, and intestinal inflammations [1, 3]. Recent research has confirmed the antioxidant, antimicrobial, antihyperglycemic, antihyperlipidemic, antihypertensive, cardioprotective, and cytotoxic activities of PS seeds. These beneficial effects are attributed to the presence of several bioactive secondary metabolites, including flavonoids, phenolic acids, cinnamyl phenols, lectins, proteins, peptides, polysaccharides, and saponins. PS seeds also constitute a well-known source of various types of nutrients including proteins, soluble and insoluble fibers, complex carbohydrates, folate, vitamin B, as well as minerals such as calcium, potassium, and iron [4].

The primary wastes generated from PS processing include peels, leaves, and stems [5, 6]. PS seeds are located inside pods and peels are typically discarded as waste. Approximately, 22 million tons of PS seeds are produced annually around the world. PS peels constitute 55% of the total volume of PS pods and are an abundant source of phenolic and bioactive natural dietary antioxidants, along with dietary fibers, protein, and calcium [4]. They are commonly used as animal feed or serve as starting material for the production of fuel or compost [7]. Additionally, PS peel flour is reported to be incorporated in biscuit and cake preparation to impart a natural green pistachio color without adding synthetic colors [4]. Nowadays, new strategies for the effective valorization of food waste are emerging. These strategies involve the extraction of bioactive metabolites and the development of value-added products, such as enzymes, prebiotics, and bioactive functional phytonutrients, from food waste. These bioactive or value‐added products can be further incorporated into pharmaceutical, cosmetic, or food industries, thereby generating additional income and ensuring regional food sustainability [8].

Several biological studies have explored extracts derived from various waste organs of PS. PS peels have been shown to possess antioxidant, cardioprotective, antidiabetic, cytotoxic, and antibacterial properties [9–11], while leaves and roots have exhibited antioxidant activity [12–14]. Various phytochemical classes, including different types of flavonoids such as flavonols, flavones, isoflavones, chalcones, and anthocyanins, along with phenolic acids and pterocarpans, have been isolated from aerial parts, leaves, shoots, flowers, and roots [15].

Liquid chromatography-mass spectrometry (LC-MS) analysis of PS seeds has revealed the presence of phenolic acids, flavonoids and amino acids [14, 16], whereas only a limited number of phenolic acids, flavonoids, and terpenoids have been identified in PS peels and aerial parts [3, 9, 17]. Consequently, the comprehensive metabolite profiles of common PS wastes, namely peels (PSP) and a combination of leaves and stems (PSLS), as well as their potential anticancer effects, have not yet been fully investigated, despite other biological effects have been reported on these wastes [9–13]. Our study will focus on breast cancer, the most prevalent cancer type among women, second only to lung cancer as the leading cause of death worldwide [18, 19]. Cancer therapies often cause undesirable effects on normal cells, necessitating novel anticancer agents with high selectivity for cancer treatment [19].

Liquid chromatography-tandem mass spectrometry (LC-MS/MS) untargeted metabolomics is a large-scale analysis focused on comprehensive metabolite profiling and chemotaxonomic studies. It significantly contributes to obtaining complete metabolite profiles of natural products, detecting qualitative and quantitative differences between metabolites, and developing hypotheses to explain these differences. This may eventually lead to the identification of bioactive metabolites and the discovery of lead compounds from natural sources [20–24]. Interpreting the massive datasets generated by LC-MS/MS metabolomics requires the use of databases and literature searches. Additionally, it can be aided by molecular networking (MN) [21]. MN is a graph-based workflow that helps organize massive MS data by finding spectral similarities between structurally related precursor ions with different MS/MS fragmentation patterns. MN compares the MS/MS spectra of different ions in a sample and organizes them based on similarities. Many free web-based platforms, such as MetaboAnalyst and the Global Natural Products Social Molecular Networking (GNPS-MN) platform can be utilized for MN. This approach holds promise for metabolite identification, dereplication, and comprehensive analysis of natural products [20–23].

Network pharmacology presents a novel strategy for drug discovery. It facilitates the identification of new bioactive agents from natural products by studying the intricate relationships among drugs, targets, and diseases, as well as the associated pathways and underlying mechanisms of action. This approach is particularly beneficial for complex diseases, including cancer, neurological disorders, and cardiovascular diseases, which involve various pathways, genes, and functional proteins. Consequently, optimized combinations of multiple drugs or drugs having multiple targets, such as plant extracts, could be disclosed to synergistically target different pathways, leading to improved curative effects [25].

In this study, the detailed metabolite profiles of the 70% ethanolic extracts of PSP and PSLS were investigated using liquid chromatography-electrospray ionization quadrupole time-of-flight-tandem mass spectrometry (LC-ESI-QTOF-MS/MS, simplified as LC-MS/MS) untargeted metabolomics followed by MN. The potentially active metabolites, key targets, and the pathways involved in cytotoxic activity against breast cancer were determined through network pharmacology. The efficacy and safety of both extracts and several polyphenolics isolated from the PSLS extract were further investigated in human breast adenocarcinoma (MCF-7) and human mammary epithelial (MCF-10a) cell lines. Our findings will contribute to the valorization of PS waste through the development of effective natural anticancer agents with high safety margins and low incidence of undesirable effects.

## Materials and methods

### Cell lines, chemicals, and reagents

Solvents used in LC-MS/MS analysis, namely acetonitrile, methanol, formic acid (≥ 95.0%), and water, were of LC-MS grade and supplied by Merck (Darmstadt, Germany). Deuterated methanol (CD_3_OD, Sigma-Aldrich, St. Louis, USA) was used as solvent for NMR analysis. All other solvents and reagents, used for the extraction and fractionation of PS wastes, including ethanol, distilled water, *n*-hexane, dichloromethane, ethyl acetate, *n*-butanol, and methanol, were of analytical grade and purchased from Piochem (6th of October city, Giza, Egypt). MCF-7 and MCF-10a cell lines were supplied by the American Type Culture Collection (Manassas, USA) and maintained frozen in liquid nitrogen at -180 °C. The 3-(4,5- dimethylthiazol-2-y1)-2,5-diphenyl tetrazolium bromide (MTT) assay kit and the reference anticancer drug, doxorubicin, were provided by Sigma-Aldrich (St. Louis, USA).

### Plant material

PS wastes (ripe green pods without seeds (PSP) and the combined leaves with stems (PSLS)) were collected from the National Center of Vegetable Research, Dokki, Giza, Egypt, in February 2021. Authentication of the plant material was done by Professor Abdelhaleem Abdelmogally, head of the Taxonomy Department, Agricultural Museum, Dokki, Giza, Egypt. A voucher specimen was deposited in the Herbarium of the Department of Pharmacognosy, Faculty of Pharmacy, Cairo University (No. 30.7.2022).

### Extraction

PSP and PSLS samples were individually air-dried and powdered. Five hundred grams of each powdered sample were extracted separately with 1 L of 70% ethanol using cold maceration (25ºC) with the aid of ultra-sonication (Elma Terrasonic TS-540, Bedford, UK). The extraction process was repeated 7 times, then each collective extract was evaporated to dryness at 60ºC under vacuum using a rotatory evaporator (Rotavapor^®^ R-100, Büchi, Switzerland), to yield the corresponding solid extracts. The recorded weights of the solid extracts of PSP and PSLS were 50 and 60 g, respectively (i.e. 10 and 12% mass per mass (m/m) of the original waste samples).

### LC-MS/MS untargeted metabolomics

Samples were prepared by individually dissolving 10 mg of each of the solid extracts, previously prepared (Sect. 2.3), in 1 mL of methanol with the aid of ultra-sonication. LC-MS/MS experiments were performed in a 1260 Infinity liquid chromatograph coupled with an orthogonal ESI interface to a 6546 LC/QTOF mass spectrometer (Agilent Technologies, Waldbronn, Germany). A Zorbax SB-C18 column (150 mm total length (L_T_) × 2.1 mm internal diameter (ID), 5 μm particle size, 90 Å pore diameter, Agilent Technologies) was used for the separations. The chromatographic, MS, and MS/MS conditions were as described in a previous work [26]. Briefly, a gradient elution using mobile phase solvents, (A) water and (B) acetonitrile (both with 0.1% (v/v) of formic acid). The optimized elution was achieved through a gradient of 5% (v/v) of solvent B for 1 min, then raising the percentage of solvent B from 5 to 95% (v/v) in 15 min, followed by cleaning and re-equilibration with 95% (v/v) of solvent B for 2 min, then from 95 to 5% (v/v) of solvent B in 2 min, and finally 5% (v/v) of solvent B for 5 min. Injection volume was 5 µL and flow rate was 350 µL/minute. Experiments were done in triplicate at room temperature. Data acquisition and processing were performed with the ChemStation LC3D software (Agilent Technologies). The obtained raw data files were converted using MS Convert 3.0 open-source software (www.proteowizard.org) to mzXML files, which were then imported to MZmine 2.53 open-source software (https://github.com/mzmine/mzmine2/releases/tag/v2.53) for peak picking, deconvolution, deisotoping, alignment, and formula prediction. Detected metabolites were characterized by their accurate molecular monoisotopic mass, retention time (t_r_), and MS/MS spectra. Metabolite identification was done by comparison with reference literature and online databases (e.g., KEGG, https://www.genome.jp/kegg/compound/ and PubChem, https://pubchem.ncbi.nlm.nih.gov/). Identities were further confirmed by MN as will be discussed in the upcoming sections. Therefore, annotated metabolites were identified at a high confidence level (Level 2a: probable structure, MS, MS/MS, and library/bibliography search) [27]. Examples of MS/MS fragmentation patterns of selected metabolites from each class are represented by Figures S1-S23.

### Molecular networking

The converted mzXML files were uploaded, using WinSCP cross-platform, to the GNPS server (https://gnps.ucsd.edu/ProteoSAFe/static/gnps-splash.jsp). The parameters for network generation were set as mentioned by Hamed et al. [28] except that the minimum pairs cosine and the minimum matched fragment ions were set to 0.65 and 3, respectively. The created MN and parameters for the negative and positive ESI modes can be accessed through the following links: https://gnps.ucsd.edu/ProteoSAFe/status.jsp?task=f56500ffb179472bb8b7d3b3098925e8 and https://gnps.ucsd.edu/ProteoSAFe/status.jsp?task=aa58b89c813a4dc38e1e72c37acf670d, respectively. The output network was merged to Feature Based Molecular Network (FBMN) and imported to the open-source software platform, Cytoscape 3.9.1 software (https://cytoscape.org/download.html) for network visualization and interpretation.

### Network pharmacology study of potential therapeutic activity against breast cancer

#### Construction of compound-target-disease network

The respective Simplified Molecular Input Line Entry Specification (SMILES) formulas of metabolites identified through LC/MS-MS untargeted metabolomics were generated and used for screening of compound-target interactions. Protein targets for the identified metabolites were extracted from two databases, SwissTargetPrediction (http://www.swisstargetprediction.ch/) and Similarity Ensemble Approach (SEA) (https://sea.bkslab.org/). The extracted protein targets were combined, dereplicated, and code-unified using UniProt Retrieve/ID mapping (https://www.uniprot.org/uploadlists/). Disease targets were determined using GeneCards (https://www.genecards.org/) and DisGeNet (https://www.disgenet.org/home/) databases. “Breast cancer” and “breast adenocarcinoma” were used as search terms. Then the identified genes were assembled and dereplicated. A Venn’s diagram was generated using Venny (https://bioinfogp.cnb.csic.es/tools/venny/) to show the common targets between compound-target and disease-target pairs. Cytoscape 3.9.1 software was used for visualizing and interpreting the compound-target-disease network.

#### Protein-protein interaction (PPI) network

The aforementioned common targets were imported to the STRING database (https://string-db.org/) to reveal the PPI. Confidence limit was set to ≥ 0.7.

#### Enrichment of gene ontology (GO) terms and kyoto encyclopedia of genes and genome (KEGG) pathways

Functional analysis and enrichment of the gene targets were performed utilizing the online tool ShinyGO 0.77 and its integrated tools (http://bioinformatics.sdstate.edu/go77/).

### Isolation of phenolic components from PSLS

Fifty grams of the solid extract of PSLS were fractionated using solvents with increasing polarities, namely *n*-hexane, dichloromethane, ethyl acetate, and *n*-butanol (10 times × 300 mL each). The ethyl acetate fraction was evaporated under vacuum at 60^o^C resulting in 3 g of the dried fraction, which was then suspended in 30 mL of distilled water and subjected to column chromatography on a diaion^®^ HP-20 column (45 g, 35 cm L_T_ × 2.5 cm ID, Merck, Darmstadt, Germany). Elution started with 500 mL of distilled water. Then, the percentage of methanol was increased by 25% increments in each step. The resulting five sub-fractions, namely water, 25%, 50%, 75%, and 100% methanol (500 mL each) were analyzed by thin layer chromatography (TLC). Then they were evaporated under vacuum at 60^o^C, weighed, and kept in a desiccator at room temperature for further analysis. The 75% methanol sub-fraction (100 mg) was dissolved in a 9:1 mixture of methanol and water and subjected to column chromatography on a Sephadex^®^ LH-20 (8 g, 28 cm L_T_ × 1.5 cm ID, Merck, Darmstadt, Germany) using isocratic elution with 90% methanol. Fractions of 2 mL were collected and analyzed by TLC. Fractions 12 and 13 were pooled and evaporated under vacuum at 50^o^C resulting in 10 mg of component 1. Similarly, fractionation by column chromatography of the 50% sub-fraction (137 mg) on the Sephadex^®^ LH-20 column using isocratic elution with 50% methanol resulted in the isolation of components 2 and 3 (5 and 9 mg, respectively). Schematic representation of the steps of extraction, fractionation, and isolation to obtain these components is represented in Figure S24. The structures of the isolated phenolic components were elucidated by nuclear magnetic resonance (NMR), using a U28-E04 Bruker Ascend 400 MHz NMR spectrometer (Karlsruhe, Germany). The NMR spectra were compared to those found in reference literature for confirmation of their identities [29, 30].

### MTT cytotoxicity assay

The MTT assay is a colorimetric method used for measurement of cell proliferation, activity, and viability. It relies on the ability of mitochondrial dehydrogenase enzymes within living cells to transform the color of 3-(4,5- dimethylthiazol-2-y1)-2,5-diphenyl tetrazolium bromide (MTT) from yellow to purple. This color change serves as an indicator of cell viability [31]. To assess the in-vitro cytotoxic activity against MCF-7 and MCF-10a cell lines of PSP and PSLS ethanolic extracts, as well as the isolated phenolic components from PSLS extract and the reference drug doxorubicin, the MTT assay was conducted as described in a previous study [31]. The presence of viable cells was visualized through the development of purple color. The optical density (OD) at 595 nm was measured using a ROBONIK P2000 ELISA reader (Robonik India Pvt Ltd, Morivali, India), and the %viability was calculated at different concentration levels using the following equation:

$$\:\text{\%}\text{V}\text{i}\text{a}\text{b}\text{i}\text{l}\text{i}\text{t}\text{y}=\:\frac{\text{O}\text{D}\:\text{o}\text{f}\:\text{s}\text{a}\text{m}\text{p}\text{l}\text{e}}{\text{O}\text{D}\:\text{o}\text{f}\:\text{c}\text{o}\text{n}\text{t}\text{r}\text{o}\text{l}\:\left(\text{n}\text{o}\:\text{d}\text{r}\text{u}\text{g}\right)}\:\times\:100$$

The half-maximal inhibitory concentration (IC_50_) values were obtained for each sample after plotting the %viability against the concentration.

## Results

### LC-MS/MS untargeted metabolomics

**Fig. 1:**
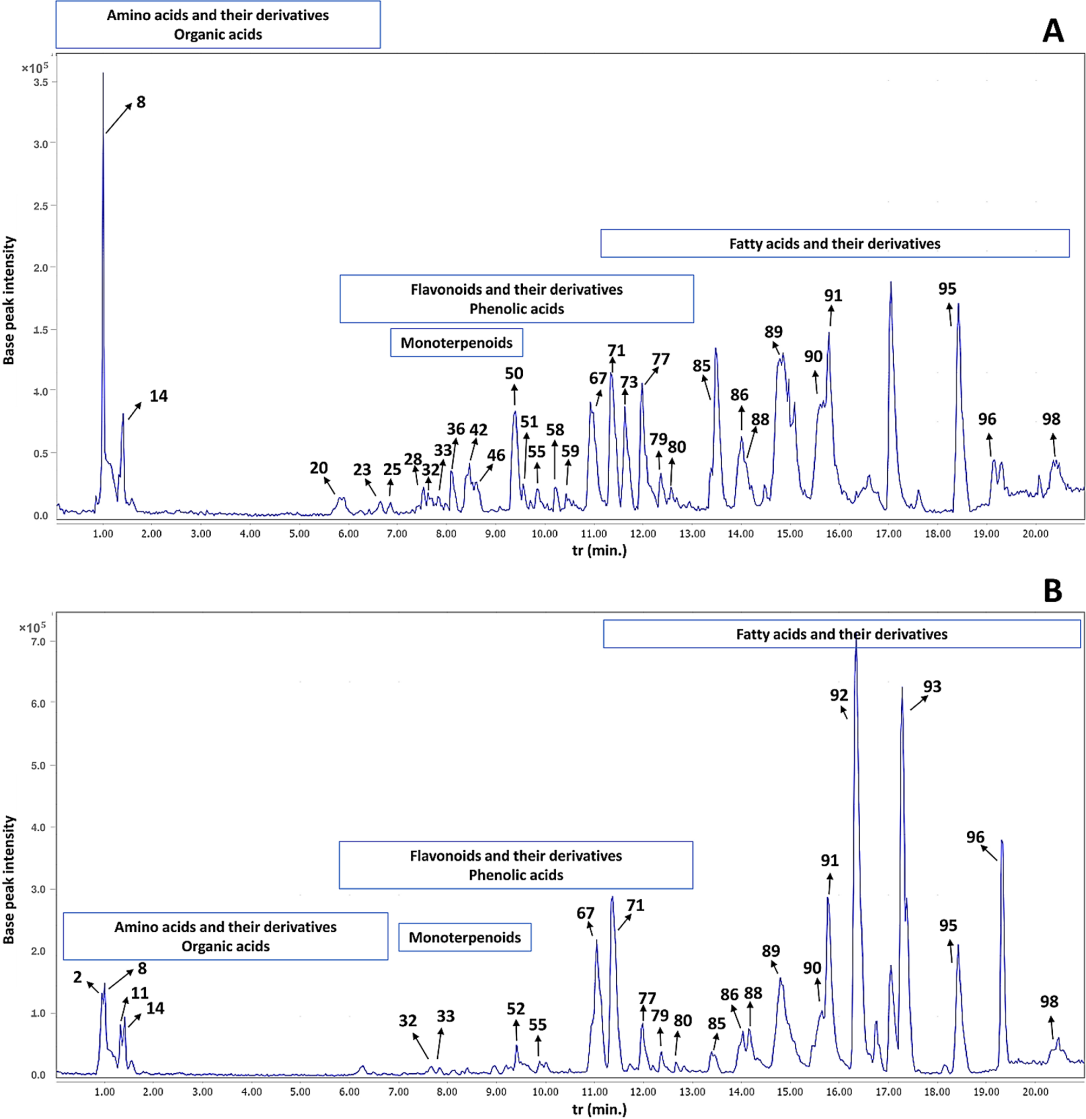
Base peak chromatograms of *Pisum sativum* L. (green pea, PS) waste extracts in the negative ESI mode. (**A**) PSLS (leaves and stems extract) and (**B**) PSP (peels extract). Metabolite numbers are as listed in Table 1

**Fig. 2:**
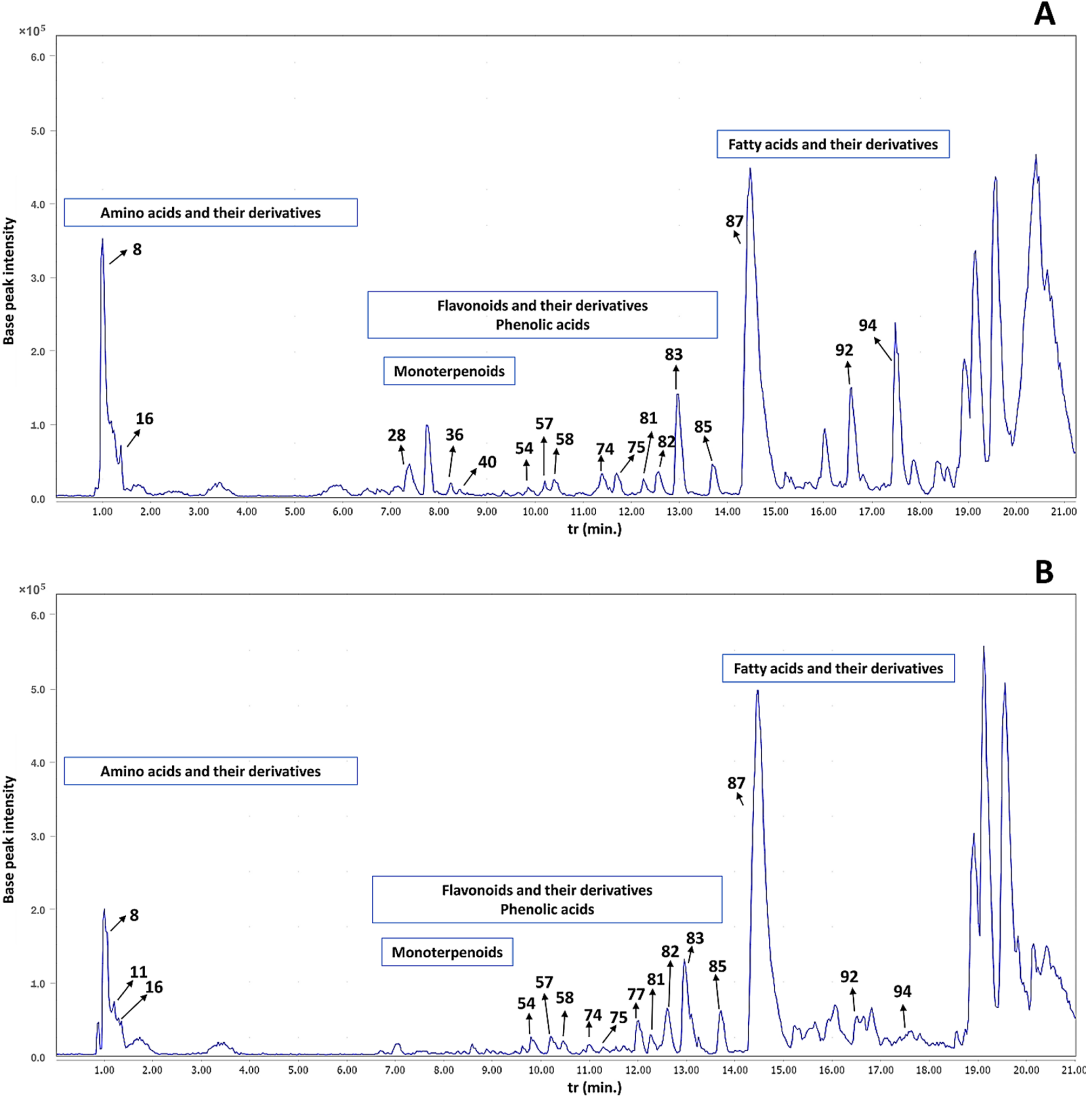
Base peak chromatograms of *Pisum sativum* L. (green pea, PS) waste extracts in the positive ESI mode. (**A**) PSLS (leaves and stems extract) and (**B**) PSP (peels extract). Metabolite numbers are as listed in Table 1

The LC-MS/MS untargeted metabolomic study revealed the presence of several classes of secondary metabolites including flavonoids and their derivatives, phenolic acids, amino acids and their derivatives, organic acids, fatty acids and their derivatives, monoterpenoids, and other miscellaneous compounds. A total of 98 metabolites were identified in PSP and PSLS extracts, including 39 flavonoids and their derivatives, 9 phenolic acids, 14 amino acids and their derivatives, 6 organic acids, 18 fatty acids and their derivatives, 4 monoterpenoids, and 8 other miscellaneous compounds. Metabolite identities, t_r_, mass to charge ratios (*m/z*) of the detected molecular ions and fragment ions, and the predicted molecular formulas are summarized in Table 1. The base peak chromatograms of PSP and PSLS extracts in the positive and negative ionization modes are depicted in Figs. 1 and 2, respectively.

**Table 1:** Metabolites identified in *Pisum sativum* L. (green pea, PS) waste extracts by LC-MS/MS untargeted metabolomics in negative and positive ESI modes

Peak	t_*r*_ (min.)	Molecular ion (M-H)^−^(m/z)	MS/MS fragments (m/z) Negative mode	Molecular ion (M + H)^+^ (m/z)	MS/MS fragments (m/z) Positive mode	Molecular Formula	Identification	Extract		Ref.
								PSLS	PSP	
Flavonoids and their derivatives										
Flavonols										
33	7.83	461.1089	285			C_21_H_18_O_12_	Kaempferol-*O*-hexuronide	+	+	[[32], 33]
37	8.24	593.1504	447, 285, 283, 253*			C_27_H_30_O_15_	Kaempferol-*O*-neohesperidoside	+	-	
42	8.62	577.1559	431, 285*, 283, 255	579.1730	287	C_27_H_30_O_14_	kaempferol-3,7-*O*-di deoxyhexoside	+	-	
43	8.63	933.2301	787, 625, 463, 413, 301, 300*, 255			C_42_H_46_O_24_	Quercetin-*O*-coumaroyl sophorotrioside	+	(+)	
44	8.64	963.2385	787, 623, 463, 343, 301, 300*, 255			C_43_H_48_O_25_	Quercetin-*O*-feruloyl sophorotrioside	+	(+)	
45	8.88	947.2451	786, 771, 609, 542, 448, 383, 285*, 283, 255			C_43_H_48_O_24_	Kaempferol-*O*-feruloyl sophorotrioside	+	(+)	
46	8.92			433.1138	287*, 271, 153, 99	C_21_H_20_O_10_	Kaempferol-*O*-deoxyhexoside	+	-	
47	8.94	917.2339	867, 771, 609, 447, 429, 312, 285*, 198			C_42_H_46_O_23_	Kaempferol-*O*-coumaroyl sophorotrioside	+	(+)	
48	9.09	447.1293	365, 285*, 270, 161, 123			C_21_H_20_O_11_	Kaempferol-*O*-hexoside	+	(+)	
63	10.60	269.0464	269*, 225, 211, 185, 59	271.0601	192*, 176, 98	C_15_H_10_O_5_	Galangin	+	+	[34]
Flavones										
34	7.91	563.1404	563, 473, 443, 383*, 353, 282, 238, 208, 165, 143, 89			C_26_H_28_O_14_	Apigenin-6-*C*-pentoside-8-*C*-hexoside	+	-	35–37
35	8.04	491.1201	329, 314*, 299, 271, 189, 123			C_23_H_24_O_12_	Tricin-*O*-hexoside	+	-	
36	8.20	577.1559	577, 487, 473, 439, 413, 395, 383, 365, 353*, 282, 221, 170, 91	579.1708	443, 409, 379*, 309, 294, 271, 119	C_27_H_30_O_14_	Apigenin-6-*C*-deoxyhexoside-8-*C*-hexoside	+	-	
54	9.81	475.1247	475, 343, 239*, 187, 125	477.1413	315*, 300, 272, 127, 85	C_23_H_24_O_11_	Cirsimarin	+	-	
56	9.94	447.1293	447, 285*, 270, 137			C_21_H_20_O_11_	Luteolin-*O*-hexoside	+	(+)	
60	10.53	269.0464	269*, 240, 223, 195, 169, 135, 121, 109, 93	271.0600	271*, 215, 161, 137, 121, 105, 88	C_15_H_10_O_5_	7,3’,4’-Trihydroxyflavone	+	+	
62	10.59	299.0563	284, 271, 255*, 227, 211, 185, 175, 149, 135, 123, 108, 81, 59			C_16_H_12_O_6_	Chrysoeriol	+	(+)	
73	11.44	537.0823	451, 417, 385, 375*, 309, 257, 203			C_30_H_18_O_10_	3,8’-Biapigenin	+	-	
75	11.64	313.0719	313, 298, 283, 270, 255, 239, 226*, 211, 183, 167	315.0873	315*, 300, 272, 255, 227, 167	C_17_H_14_O_6_	Cirsimaritin	+	(+)	
79	12.31	297.0775	282*, 267, 254, 239, 151, 93, 73			C_17_H_14_O_5_	3’,7-Dimethoxy-3-hydroxyflavone	+	(+)	
88	14.54	269.0456	269*, 254, 225, 220, 195, 179, 105			C_15_H_10_O_5_	Baicalein	+	(+)	
Flavanones										
28	7.56	323.1248	292, 282, 229, 203, 189*, 175, 161, 146, 138, 97, 71, 59	325.1405	325, 271, 250, 215, **191**, 173, 69	C_20_H_20_O_4_	6-Prenyl pinocembrin	+	(+)	[38]
39	8.45	549.1619	549, 417, 255*, 135, 119, 89, 59			C_26_H_30_O_13_	Liquiritigenin-*O*-pentosyl hexoside	+	-	[39]
41	8.58	417.1194	255, 149, 135*, 119			C_21_H_22_O_9_	Liquiritigenin-*O*-hexoside	+	-	
58	10.44	255.0665	255, 135, 119*, 91	257.0815	257, 239, 168, 147, **137**, 93	C_15_H_12_O_4_	Liquiritigenin	+	-	
69	11.18	271.0977	255, 226, 184, 165*, 150, 137, 123, 108, 93, 80			C_15_H_12_O_5_	Naringenin	+	(+)	
74	11.53			287.0924	269, 255, 241, 227, 209, **153**, 123, 110	C_16_H_14_O_5_	Isosakuranetin	(+)	+	
82	12.94	269.0822	254*, 221, 210, 135, 121, 119, 98	271.0979	271, 241, 210, 177, 137*, 109, 79	C_16_H_14_O_4_	7-methyl liqueritigenin	+	(+)	
Isoflavones										
57	10.21	253.0505	253*, 224, 197, 179, 135, 117, 91	255.0652	255*, 237, 199, 150, 137, 131, 118	C_15_H_10_O_4_	Daidzein	+	+	[40]
59	10.50	283.0613	268*, 239, 211, 148, 120			C_16_H_12_O_5_	5-methyl genistein	+	(+)	
64	10.88			285.0765	285, 270*, 253, 225, 183, 169, 157, 134	C_16_H_12_O_5_	Calycosin	(+)	+	
66	11.00			315.0874	315*, 300, 282, 255, 240, 199, 167, 139, 119	C_17_H_14_O_6_	Dipteryxin	+	(+)	
72	11.39	269.0464	269*, 254, 225, 201, 169, 133, 107			C_15_H_10_O_5_	Genistein	+	(+)	[40]
78	12.16	267.0664	267, 252*, 223, 208, 195, 132	269.0816	269*, 253, 226, 213, 198, 185, 155, 136	C_16_H_12_O_4_	7-methyl daidzein	+	(+)	
81	12.60			299.0922	299*, 284, 243, 211	C_17_H_14_O_5_	5,7-Dimethyl genistein	+	(+)	
84	13.08			283.0973	283*, 268, 240, 212, 197, 181, 161, 148, 121, 91	C_17_H_14_O_4_	7,4’-Dimethoxy isoflavone	+	(+)	
85	13.36	283.0611	268*, 239, 211, 195, 183, 168, 148, 120	285.0765	285*, 270, 257, 242, 229, 167	C_16_H_12_O_5_	Prunetin	+	(+)	
Chalcones										
65	10.89	269.0822	269*, 253, 241, 225, 212, 193, 181, 161, 148, 136, 118, 108, 92	271.0979	271, 241, 177, 161, **137**, 109	C_16_H_14_O_4_	4,4’-dihydroxy-2’-methoxychalcone	+	(+)	[41]
77	11.98	255.0663	255, 213, 149, 135, 119*, 91	257.0808	257*, 165, 137, 90, 68	C_15_H_12_O_4_	Isoliquiritigenin	+	(+)	
**Phenolic acids**										
23	6.66	165.0558	164, 150, 121, 107, 93*, 80, 52			C_9_H_10_O_3_	Hydroxyphenyl propanoic acid	+	-	[42]
25	6.75	153.0194	153, 109, 108*, 96, 81, 55			C_7_H_6_O_4_	Gentisic acid	(+)	+	
32	7.64	153.0194	153, 109, 108*, 96, 81, 55			C_7_H_6_O_4_	Protocatechuic acid	(+)	+	
40	8.54	163.0401	163, 119*, 95	165.0551	147*, 119, 91, 65	C_9_H_8_O_3_	*p*-Coumaric acid	+	(+)	
53	9.75	181.0509	181, 109, 108*, 93, 69			C_9_H_10_O_4_	Veratric acid	+	(+)	
55	9.89	137.0243	93*, 65			C_7_H_6_O_3_	Salicylic acid	(+)	+	[42]
61	10.57	151.0402	107*, 92, 65			C_8_H_8_O_3_	*p*-Hydroxyphenyl acetic acid	+	+	
80	12.57	193.0870	193*, 177, 121, 89, 74			C_10_H_10_O_4_	Ferulic acid	+	+	
83	12.97			181.1221	181, 163, 145, 135*, 121, 107, 91, 79	C_9_H_8_O_4_	Caffeic acid	+	(+)	
**Amino acids and their derivatives**										
1	0.93	118.0510	118, 100, 74*, 56	120.0652	74, 56*	C_4_H_9_NO_3_	Threonine	(+)	+	[43]
2	0.95	132.0300	115, 100, 88*, 79, 71, 59			C_4_H_7_NO_4_	Aspartic acid	+	(+)	
4	0.97			175.0584	175, 130, 112, 70*	C_6_H_14_N_4_O_2_	Arginine	+	(+)	
7	1.02	114.0563	114*, 83, 70, 56, 50	116.0709	116, 70*	C_5_H_9_NO_2_	Proline	(+)	+	
8	1.04	118.0510	118, 99*, 74, 72, 60	120.0657	74, 56*	C_4_H_9_NO_3_	Homoserine	+	(+)	
9	1.05	290.0881	212, 200, 170, 128*, 101, 84, 70			C_11_H_17_NO_8_	N-Fructosyl pyroglutamate	(+)	+	
10	1.06	160.0616	160, 142, 118, 98, 74*, 58			C_6_H_11_NO_4_	O-acetyl homoserine	(+)	+	
11	1.30	116.0716	99, 92, 78, 75*	118.0868	118*, 72, 55, 58	C_5_H_11_NO_2_	Valine	+	+	
15	1.60	130.0874	130*, 117, 83, 69, 58	132.1022	86*, 69, 58	C_6_H_13_NO_2_	Leucine	+	+	
16	1.67	180.0666	172, 163, 157, 120, 115, 93*, 72	182.0817	165, 147, 136, 119*, 91	C_9_H_11_NO_3_	Tyrosine	+	+	
17	1.77			132.1025	86*, 73, 69	C_6_H_13_NO_2_	Isoleucine	+	+	
18	2.57	164.0717	147, 103*, 77, 72			C_9_H_11_NO_2_	Phenylalanine	+	+	
21	6.25	203.0830	186, 159, 142, 116*, 72	205.0975	188, 170, 160, 146*, 132, 118, 89, 56	C_11_H_12_N_2_O_2_	Tryptophan	+	+	
31	7.62	172.0978	130*, 82			C_8_H_15_NO_3_	N-acetyl leucine	(+)	+	
**Organic acids**										
3	0.96	195.0511	195, 177, 159, 141, 129, 99, 87, 75*, 59			C_6_H_12_O_7_	Gluconic acid	(+)	+	[44]
5	0.99	135.0295	135, 87, 75*, 59			C_4_H_8_O_5_	Threonic acid	+	+	
12	1.41	191.0195	129, 111*, 87, 67, 57			C_6_H_8_O_7_	Citric acid	+	(+)	
13	1.48	117.0195	**73**			C_4_H_6_O_4_	Succinic acid	+	(+)	
14	1.57	161.0456	99, 59, 57*			C_6_H_10_O_5_	3-Hydroxy-3-methylglutaric acid	(+)	+	
22	6.28	175.0613	115*, 85, 69, 59			C_7_H_12_O_5_	2-Isopropylmalic acid	(+)	+	
**Fatty acids and their derivatives**										
29	7.60	255.1241	255, 237, 240, 211, 182*, 167, 149, 138, 97, 57			C_13_H_20_O_5_	Tricladic acid A	+	(+)	[44, 45]
30	7.61	433.2085	271, 234*, 207, 159, 95, 75			C_20_H_34_O_10_	Peroxydisebacic acid	+	(+)	
51	9.40	187.0978	187, 169, 143, 125*, 97, 57			C_9_H_16_O_4_	Azelaic acid	+	+	
67	11.06	327.2175	327, 309, 291, 283, 269, 251, 229, 211, 183*, 171, 125, 97, 85, 71, 57			C_18_H_32_O_5_	9,12,13-trihydroxyoctadeca-10,15-dienoic acid	(+)	+	
68	11.14	215.1287	197*, 171, 153, 134, 102			C_11_H_20_O_4_	Undecanedioic acid	+	+	
71	11.35	329.2335	311, 293, 229, 211*, 183, 171, 155, 139, 127, 99			C_18_H_34_O_5_	5,8,11-trihydroxyoctadec-9-enoic acid	(+)	+	
86	14.04	313.2384	313, 295, 277, 269, 195, 183*, 129, 99, 58			C_18_H_34_O_4_	Octadecanedioic acid	(+)	+	[44, 45]
87	14.45			274.2752	274*, 256, 106, 88, 70, 57	C_16_H_35_NO_2_	Lauryldiethanolamine	+	+	
89	14.98	275.2017	275, 257, 231, 218, 167, 106*, 101, 71, 59			C_18_H_28_O_2_	Stearidonic acid	+	(+)	
90	15.77	295.2278	295, 277*, 251, 195, 171			C_18_H_32_O_3_	9-hydroxy-10,12-octadecadienoic acid	-	+	
91	16.36	297.2438	297, 279, 253, 185*			C_18_H_34_O_3_	12-Hydroxyoctadec-9-enoic acid	-	+	
92	16.59			281.2483	245, 133, 109, 95, 83, 69*, 55	C_18_H_32_O_2_	Chaulmoogric Acid	-	+	
93	17.29	299.2594	299*, 281, 255, 253, 141			C_18_H_36_O_3_	12-Hydroxyoctadecanoic acid	-	+	
94	17.49			283.2644	135, 121, 107, 97, 83, 69, 57*	C_18_H_34_O_2_	Petroselinic acid	-	+	
95	18.44	271.2279	271, 225*, 227			C_16_H_32_O_3_	Hydroxy palmitic acid	+	+	
96	19.31	279.2331	279*, 261, 235, 96			C_18_H_32_O_2_	Linoleic acid	(+)	+	
97	19.68	313.2752	313, 267*, 251, 171, 57			C_19_H_38_O_3_	Hydroxy nonadecanoic acid	+	(+)	
98	20.48	281.2490	281*, 237, 242, 98			C_18_H_34_O_2_	Oleic acid	(+)	+	
**Monoterpenoids**										
27	7.34	431.1922	333, 136, 89*, 59			C_20_H_32_O_10_	Sacranoside A	+	-	[46]
38	8.39	401.1823	401, 239, 221, 195, 177, 123, 101, 89, 85, 59*			C_19_H_30_O_9_	Cannabiside D	+	(+)	[47]
50	9.38	239.1291	238, 220, 195, 179, 158*, 125, 123, 109, 78, 57			C_13_H_20_O_4_	Aglycone of cannabiside D	+	(+)	[47]
52	9.62			197.1178	197, 161, 133*, 93, 79	C_11_H_16_O_3_	Loliolide	+	(+)	[48]
**Miscellaneous compounds**										
6	1.00	179.0563	179, 161, 95, 85, 75*, 59			C_6_H_12_O_6_	Glucose	(+)	+	[49]
19	2.60	218.1034	146, 99, 88*, 71			C_9_H_17_NO_5_	Pantothenic acid	+	+	
20	5.87	217.0720	217, 171, 155, 143, 115, 85, 72*			C_9_H_14_O_6_	Triacetin	+	-	[50]
24	6.72	109.0294	109, 108*			C_6_H_6_O_2_	Catechol	+	+	[51]
26	7.04			188.0719	188, 170, 146, 127, 118*, 85	C_11_H_9_NO_2_	Indoleacrylic acid	+	+	[49]
49	9.12	361.0570	297*, 285, 240, 227, 197, 179, 161, 148, 136, 122, 109			C_20_H_26_O_6_	Secoisolariciresinol	+	(+)	[52]
70	11.25	297.0395	297*, 269, 241, 211, 197, 181, 149, 93, 71	299.0922	299*, 284, 243, 211, 151	C_17_H_14_O_5_	Pterocarpin	+	+	[53]
76	11.96	941.5119	941*, 795, 733, 633, 615, 457, 205, 101, 59	943.5306	383, 271, 247, 203, 163, 141, 71*	C_48_H_78_O_18_	Soyasaponin Bb	+	+	[54]

+: Present, (+): Present in lower abundance, -: Absent, *: Base peak (“The most intense peak”), PSLS: *Pisum sativum* leaves and stems extract, PSP: *Pisum sativum* peels extract

### MN study

**Fig. 3:**
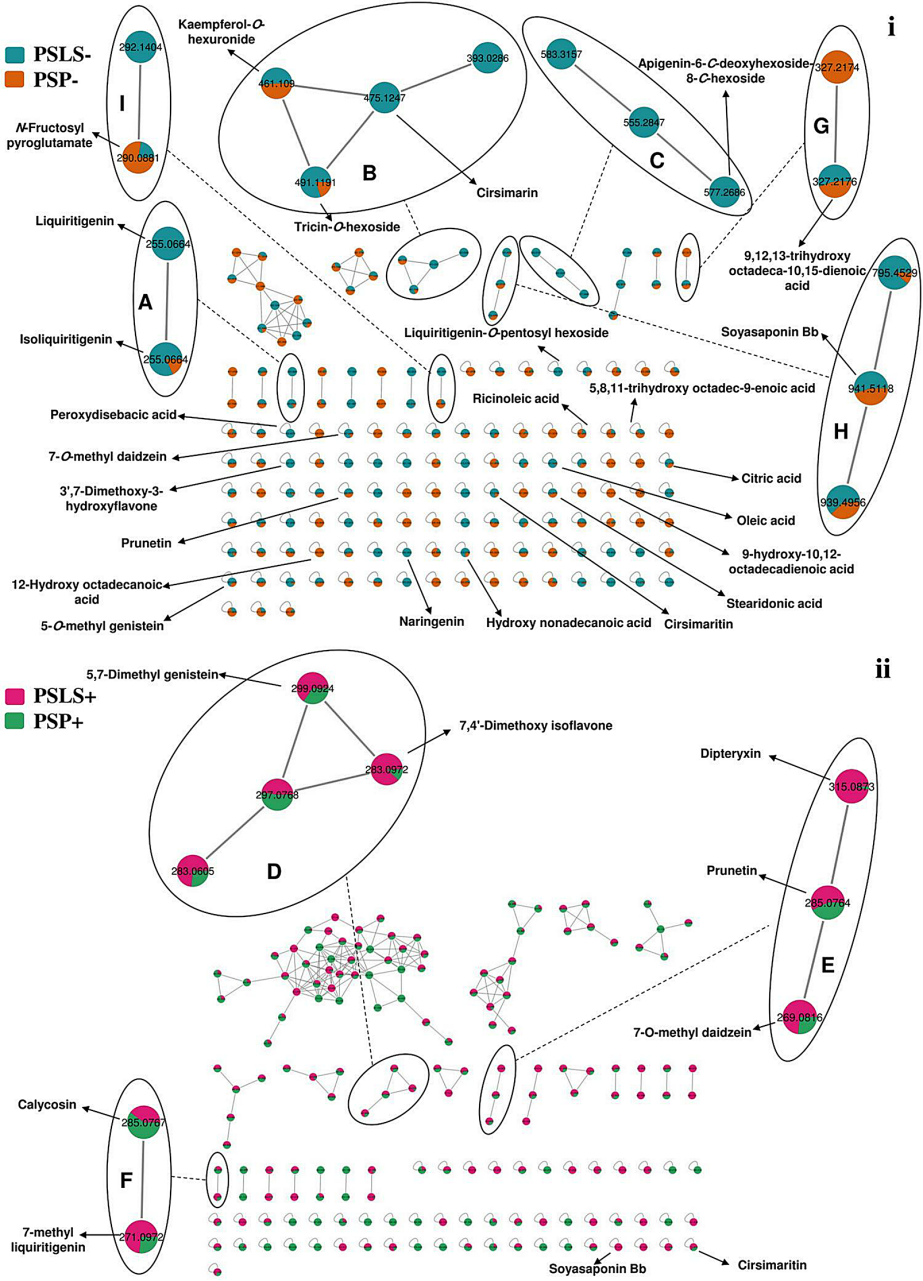
Molecular network (showing clusters of metabolites of interest) based on LC-MS/MS data in *Pisum sativum* L. (green pea, PS) wastes. (i) Negative ESI mode and (ii) Positive ESI mode. Clusters **A** to **F**: flavonoids and their derivatives, **G**: fatty acids and their derivatives, **H**: saponins, **I**: amino acid and their derivatives. The network is displayed as a pie chart to reflect the relative abundance of each metabolite precursor ion in the analyzed samples

MN was used to further confirm the metabolite identification in PS samples. In this approach, molecules are grouped into clusters based on common fragmentation patterns. Molecules with similar fragmentation patterns are interconnected, while those with dissimilar patterns are separated. Molecules that do not form groups are represented as single nodes. The color of network nodes corresponds to the sample type and is labeled with the precursor ion *m/z* values. Nodes are displayed as pie charts to indicate the relative abundance of detected molecular ions in the investigated samples [20–23]. The MN study results are represented in Fig. 3.

### Isolation of phenolic components from PSLS

In order to get a complementary insight on the PSLS extract composition, different phenolic components were isolated from its ethyl acetate fraction. NMR analysis of the isolated components led to the identification of component 1 as a mixture of two closely related flavanones (liquiritigenin and 7-methyl liquiritigenin). Components 2 and 3 were identified as trans *p*-coumaric acid, and methyl cis *p*-coumarate, respectively [29, 30]. The structures of the isolated components are represented by Figure S25. Figures S26-S34 represent the NMR spectra of the isolated components.

### Network pharmacology study of potential therapeutic activity against breast cancer

Network pharmacology functions as a comprehensive in-silico approach that constructs a “protein–compound/disease–gene” network. This aids in revealing the underlying mechanisms and synergistic actions of plant extracts, shifting the paradigm from “one-target, one-drug” to a “network-target, multiple-component-therapeutics” [55]. The identified metabolites were investigated through network pharmacology to discern the key metabolites associated to breast cancer protein targets and genes, as well as to elucidate their potential mechanisms of action.

#### Compound-target-disease network

**Fig. 4:**
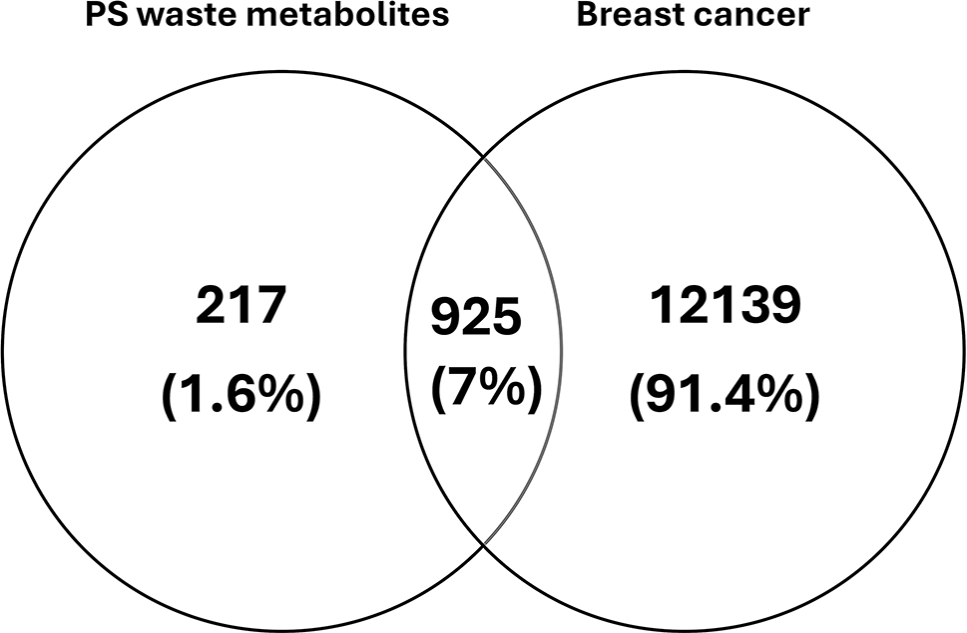
Venn’s diagram representing the intersection of protein targets common to both the identified metabolites in *Pisum sativum* L. (green pea, PS) wastes and breast cancer

Potential protein targets for all the identified metabolites in both PS waste extracts were retrieved from the SwissTargetPrediction and SEA databases. The potential protein targets linked to breast cancer were also determined. Venn’s diagram of Fig. 4 showed 925 common targets between the two domains. The common targets with the highest degree of involvement are summarized in Table S1.

**Fig. 5:**
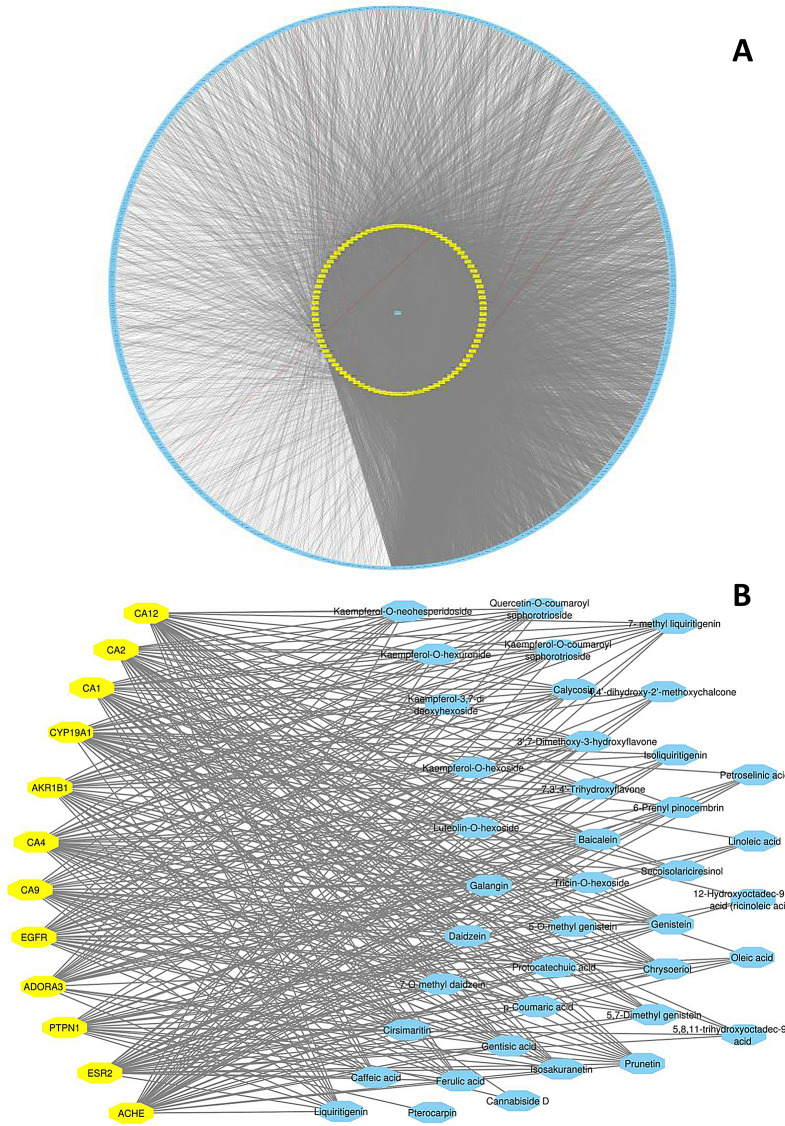
(**A**) Compound-target-disease network and (**B**) sub-network showing the key metabolites and their interactions with the key protein targets

The analysis of the constructed network (Fig. 5) revealed that flavonoids and their derivatives along with phenolic acids were the key metabolites, as they exhibited the highest degree of involvement, as shown in Table 2.

**Table 2:** Metabolites identified in *Pisum sativum* L. (green pea, PS) waste extracts by LC-MS/MS untargeted metabolomics, ranked according to the degree of involvement in the compound-target-breast cancer network

Identified metabolite	Degree	Betweenness centrality	Closeness centrality
Kaempferol-*O*-neohesperidoside **(37)**	143	0.014612	0.379599
Kaempferol-*O*-hexuronide **(33)**	132	0.014563	0.376581
Kaempferol-3,7-di deoxyhexoside **(42)**	122	0.008718	0.373878
Kaempferol-*O*-coumaroyl sophorotrioside **(47)**	97	0.001701	0.367289
Quercetin-*O*-coumaroyl sophorotrioside **(43)**	97	0.001610	0.367289
4,4’-dihydroxy-2’-methoxychalcone **(65)**	96	0.004110	0.366772
*p*-Coumaric acid **(40)**	95	0.002978	0.366772
Isoliquiritigenin **(77)**	95	0.002378	0.366772
3’,7-Dimethoxy-3-hydroxyflavone **(79)**	95	0.001493	0.366772
Protocatechuic acid **(32)**	94	0.002878	0.366514
Gentisic acid **(25)**	94	0.002591	0.366514
5,7-Dimethyl genistein **(81)**	94	0.003810	0.366514
Pterocarpin **(70)**	93	0.003866	0.366256
Secoisolariciresinol **(49)**	93	0.004012	0.365742
12-Hydroxyoctadec-9-enoic acid **(91)**	93	0.002506	0.365999
Ferulic acid **(80)**	93	0.002618	0.366256
Prunetin **(85)**	93	0.002286	0.366256
Genistein **(72)**	93	0.001700	0.365742
Calycosin **(64)**	93	0.002372	0.366256
7-Methl liquiritigenin **(82)**	93	0.003146	0.366256
6-Prenyl pinocembrin **(28)**	93	0.003302	0.366256
Chrysoeriol **(62)**	93	0.001338	0.366256
7,3’,4’-Trihydroxyflavone **(60)**	93	0.001391	0.365485
Galangin **(63)**	93	0.001402	0.366256
Oleic acid **(98)**	92	0.002182	0.365999
Petroselinic acid **(94)**	92	0.002368	0.365999
5,8,11-trihydroxyoctadec-9-enoic acid **(71)**	92	0.003067	0.365742
Caffeic acid **(83)**	92	0.002446	0.365999
7-Methyl daidzein **(78)**	92	0.003118	0.365999
5-Methyl genistein **(59)**	92	0.002063	0.365999
Daidzein **(57)**	92	0.001942	0.365485
Isosakuranetin **(74)**	92	0.002490	0.365999
Baicalein **(88)**	92	0.001738	0.365999
Luteolin-*O*-hexoside **(56)**	92	0.001566	0.365999
Kaempferol-*O*-hexoside **(48)**	92	0.001887	0.365999
Cannabiside D **(38)**	91	0.003498	0.365742
Linoleic acid **(96)**	91	0.002348	0.365742
Liquiritigenin **(58)**	91	0.002350	0.365742
Cirsimaritin **(75)**	91	0.001508	0.365742
Tricin-*O*-hexoside **(35)**	91	0.002004	0.365742

Betweenness centrality is a measure of centrality in a graph based on shortest paths, while closeness centrality is an indication of how close a node is to all other nodes in the network

Metabolite numbers are as listed in Table 1

#### PPI network

**Fig. 6:**
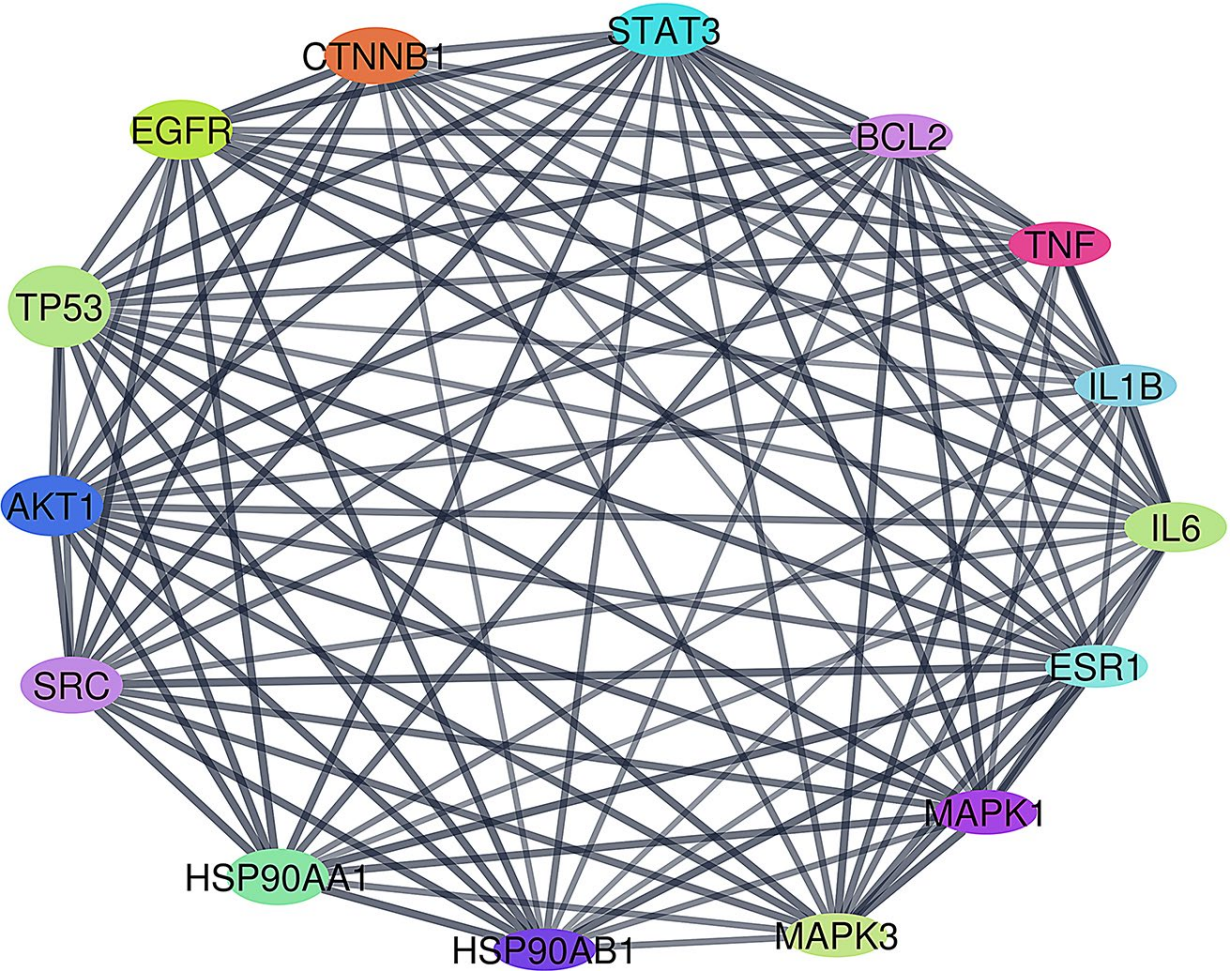
PPI network of the most interconnected targets involved in breast cancer

A PPI network is a subcellular web of nodes (proteins) connected by edges (interactions between these proteins). This approach studies the interactions between the common protein targets identified from the constructed compound-target-disease network, thus aids in gaining a global view of the investigated biological or pathological processes at both the molecular and systems level [56]. PPI network results are represented by Fig. 6 and Table S2.

#### GO and KEGG pathway enrichment analysis

**Fig. 7:**
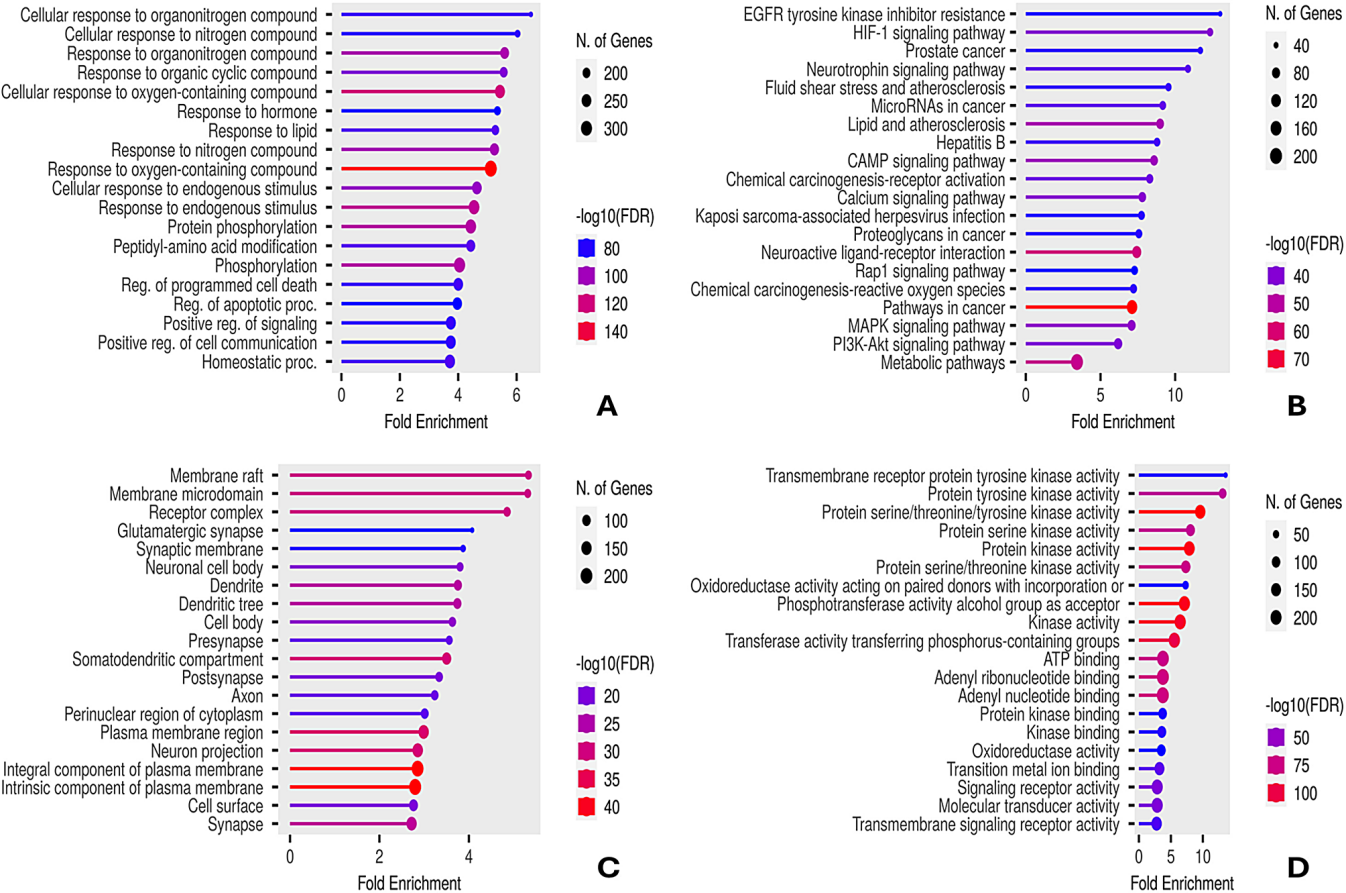
Functional analysis of the target genes identified by the compound-target-disease network. (**A**) Top GO biological processes, (**B**) top KEGG pathways, (**C**) top GO cellular components, and (**D**) top GO molecular functions. The circle size and the color density represent the number of involved genes and p-value, respectively. The fold enrichment is plotted on the abscissa while names are represented on the ordinate

The identified protein targets (Tables S1 and S2) underwent enrichment analysis, with the top 20 related functions displayed in Fig. 7. Fold enrichment, defined as the ratio between the relative frequency of genes belonging to a given pathway in the library and in the reference genome [57], indicates the extent to which genes of a particular pathway are overrepresented. The most enriched biological processes were associated with cellular response to nitrogenous and organo-nitrogenous compounds (Fig. 7A). Meanwhile, the most enriched KEGG pathways involved EGFR tyrosine kinase inhibitor resistance and the hypoxia-inducible factor 1 (HIF-1) signaling pathway (Fig. 7B). The target genes were related to membrane components (Fig. 7C), with molecular functions enriched in the binding of tyrosine kinases (Fig. 7D).

### MTT cytotoxicity assay

The MTT assay is a reliable, quantitative, and sensitive colorimetric method to measure cell viability and proliferation. Regarded as one of the simplest and most cost-effective cytotoxic assays, it requires minimal reagents and equipment. Additionally, it offers a combination of accuracy and rapid results [31]. The results of the cytotoxic assay are represented by Table 3 and Figures S31 and S32.

**Table 3:** IC_50_ values of *Pisum sativum* L. (green pea, PS) waste extracts and the isolated components from the leaves and stems (PSLS) extract on MCF-7 and MCF-10a cell lines

Extract/Isolated compound	IC_50_ µg/mL		IC_50_ µM		Selectivity index IC_50_ MCF-10a/ IC_50_ MCF-7
	MCF-7	MCF-10a	MCF-7	MCF-10a	
PSLS	17.67 ± 1.12	62.07 ± 5.86			3.51
PSP	32.92 ± 1.64	53.30 ± 3.48			1.62
Liquiritigenin (58)/7-methyl liquiritigenin (82) mixture	11.10 ± 0.46	31.26 ± 1.49			2.82
Trans *p*-coumaric acid (40)	4.49 ± 0.19	22.67 ± 1.08	28.20 ± 9.6	146.46 ± 55.24	5.05
Methyl cis *p*-coumarate	1.18 ± 0.05	32.36 ± 2.09	6.91 ± 2.73	181.97 ± 21.23	27.42
Doxorubicin	2.69 ± 0.11	14.22 ± 0.68	5.13 ± 1.62	25.93 ± 2.31	5.28

PSLS *Pisum sativum* leaves and stems extract. PSP: *Pisum sativum* peels extract. Metabolite numbers are as listed in Table 1

## Discussion

### LC-MS/MS untargeted metabolomics

In total 84 and 38 metabolites were identified in both extracts in negative and positive ESI modes, respectively. The negative ESI mode allowed the identification of a larger number of metabolites, with only 14 metabolites exclusively detected in positive ESI mode. These included some flavonoids, phenolic acids, amino acids, and fatty acids. This can be explained by the higher sensitivity of the negative ESI mode compared to positive one in the case of flavonoids and phenolic acids [58], which represented the major class of identified metabolites. Regarding the metabolite profile of both extracts, a total of 93 and 84 metabolites were identified in both ESI modes in PSP and PSLS extracts, respectively. The investigated extracts showed high similarity, with 79 metabolites detected simultaneously in both. They presented differences in certain metabolites, such as flavonoids and monoterpenoids, which had higher abundance in the PSLS extract, as can be observed from Table 1; Fig. 3.

#### Flavonoids and their derivatives

Flavonoids are plant-produced secondary metabolites responsible for protecting plants from UV radiation, attracting insects, and aiding growth and fertility [59]. They have a basic skeleton containing 15 carbons, two benzene rings (A and B) connected through a bridge of three carbons forming a heterocyclic pyran ring (C). The difference in the arrangement of these rings, as well as the attachment of various substituents to the rings result in a wide range of flavonoid subclasses [60]. Flavonols, flavones, flavanones, isoflavones, chalcones, and some corresponding derivatives, such as glycosides, acyl glycosides, and prenylated derivatives, were identified in PSP and PSLS extracts.

##### Flavonols

Kaempferol-*O*-hexuronide **(Peak 33)**, kaempferol-*O*-hexoside **(48)**, and galangin **(63)** were identified in PSP and PSLS extracts. Whereas kaempferol-*O*-neohesperidoside **(37)**, kaempferol-3,7-*O*-di deoxyhexoside **(42**, Figure S1**)**, kaempferol-*O*-deoxyhexoside **(46)** were identified only in PSLS extract. The aforementioned kaempferol derivatives showed a common fragment ion at 285 *m/z* in the negative ESI mode and/or 287 in the positive ESI mode, which represents the kaempferol aglycone. Kaempferol-*O*-hexuronide **(33)** was identified based on the loss of 176 mass units, in the negative ionization mode, which corresponds to hexuronic acid [33]. Similarly, kaempferol-*O*-hexoside **(48)** and kaempferol-*O*-deoxyhexoside **(46)** showed losses of 162 and 146 mass units, upon fragmentation, which represent *O*-hexosyl and *O*-deoxyhexosyl moities, respectively [33]. Kaempferol-*O*-neohesperidoside **(37)** (deoxyhexosyl hexoside) and kaempferol-3,7-*O*-di deoxyhexoside **(42)** were identified on the same basis. Besides, two kaempferol and two quercetin sophorotrioside acylated derivatives were detected in PSP and PSLS extracts and were identified as follows: quercetin-*O*-coumaroyl sophorotrioside **(43)**, quercetin-*O*-feruloyl sophorotrioside **(44)**, kaempferol-*O*-feruloyl sophorotrioside **(45)**, and kaempferol-*O*-coumaroyl sophorotrioside **(47)**. Upon fragmentation, the coumaroyl and feruloyl derivatives showed a loss of 146 or 176 mass units, corresponding to coumaroyl or feruloyl moieties, respectively, followed by the sequential loss of three hexosyl moieties, which confirms their identification [32]. Kaempferol glycosides and galangin **(63)** were reported to exhibit antiproliferative and apoptotic activity against breast cancer cell lines, respectively [61, 62].

##### Flavones

Five flavones were identified in both PSP and PSLS extracts. They were identified as 7,3’,4’-trihydroxyflavone **(60)**, chrysoeriol **(62**, Figure S2**)**, cirsimaritin **(75)**, 3’,7-dimethoxy-3-hydroxyflavone **(79)**, and baicalein **(88)**. Moreover, five flavone *O*-glycosides and one biflavone were identified in PSLS extract only. Tricin-*O*-hexoside **(35)** and luteolin-*O*-hexoside **(56)** showed a fragment ion peak at 329 *m/z* and 285 *m/z*, respectively, corresponding to tricin and luteolin. In the same way, cirsimarin **(54**, Figure S3 exhibited a fragment ion peak, in the positive ESI mode, at 315 *m/z*, which represents its aglycone, cirsimaritin. In addition, apigenin-6-*C*-pentoside-8-*C*-hexoside **(34**, Figure S4) and apigenin-6-*C*-deoxyhexoside-8-*C*-hexoside **(36)** were identified based on the loss of 90 and 120 mass units, the common fragmentation pattern of *C*-hexosides, in addition to 60 and 90 mass units in case of *C*-pentoside, or 74 and 104 mass units for *C*-deoxyhexoside [63]. Finally, 3,8’-biapigenin **(73)** was a biflavone exclusively identified in PSLS extract. Different flavones, including cirsimarin **(54)**, chrysoeriol **(62)**, and baicalein **(88)**, are reported to possess a potent cytotoxic activity against breast cancer cell lines [64, 65].

##### Flavanones

Three flavanones, naringenin **(69)**, isosakuranetin **(74)**, 7-methyl liquiritigenin **(82)**, along with a prenylated flavanone, 6-prenyl pinocembrin **(28)**, were identified in PSP and PSLS extracts. In addition, liquiritigenin **(58**, Figure S5) along with two liquiritigenin glycosides were identified in PSLS extract only, namely liquiritigenin-*O*-pentosyl hexoside **(39**, Figure S6) and liquiritigenin-*O*-hexoside **(41)**. Among the identified flavanones, liquiritigenin **(58)** and naringenin **(69)** were proven to be potent inhibitors of proliferation, migration, and progression of breast cancer [66, 67].

##### Isoflavones

Daidzein **(57**, Figure S7), calycosin **(64**, Figure S8), dipteryxin **(66)**, genistein **(72)**, and prunetin **(85)**, along with the methylated derivatives, 5-methyl genistein **(59)**, 7-methyl daidzein **(78)**, 5,7-dimethyl genistein **(81)**, and 7,4’-dimethoxy isoflavone **(84)**, were identified in both PSP and PSLS extracts. Isoflavones, especially daidzein **(57)** and genistein **(72)**, were shown to exhibit a potent cytotoxic activity against breast cancer cell lines [68]. In addition, the dietary consumption of isoflavones was reported to reduce the risk of breast cancer in postmenopausal women [68].

##### Chalcones

Two chalcones, 4,4’-dihydroxy-2’-methoxychalcone **(65)** and isoliquiritigenin **(77**, Figure S9), were identified in PSP and PSLS extracts. Reports concerning the effect of isoliquiritigenin **(77)** against breast cancers revealed its cytotoxic, migration inhibitory, and neoangiogenic suppressive effects [69, 70].

#### Phenolic acids

Phenolic acids are abundant in different plants as they play an important role in the plant-microbe symbiosis and plant defense against microbial attacks [71]. They consist of a phenolic ring bearing a carboxylic group along with one or more hydroxyl groups [71]. They are found in many plants either in free or bound forms, that include glycosides, esters, and amides [72]. Eight phenolic acids were identified in PSP and PSLS extracts, namely gentisic acid **(25)**, protocatechuic acid **(32)**, *p*-coumaric acid **(40**, Figure S10), veratric acid **(53)**, salicylic acid **(55)**, *p*-hydroxyphenyl acetic acid **(61)**, ferulic acid **(80)**, and caffeic acid **(83**, Figure S11). Whereas hydroxyphenyl propanoic acid **(23)** was exclusively identified in PSLS extract. Phenolic acids have a general fragmentation pattern in the negative ESI mode showing the loss of 44 mass units corresponding to a CO_2_ group [73]. Various phenolic acids, including caffeic **(83)**, ferulic **(80)**, and protocatechuic **(32)** acids, were reported to exhibit a potent growth inhibitory effect on breast cancer cell lines, possibly through the interaction with different intracellular and membrane receptors [74].

#### Amino acids and their derivatives

Amino acids are organic compounds having a central carbon atom (*α*-carbon) to which a carboxyl group (COOH), an amino group (NH_2_), and a specific side chain are attached [75]. They play several crucial roles for plants. Their major importance relies in fighting the hazardous effects of drought and salinity by changing the plant’s osmotic pressure. They also play a key role in enhancing the plant yield and quality through the production of some natural growth hormones and stimulation of cell division [75]. Eleven amino acids were identified in both PSP and PSLS extracts, specifically threonine **(1)**, aspartic acid **(2**, Figure S12), arginine **(4)**, proline **(7**, Figure S13), homoserine **(8)**, valine **(11)**, leucine **(15)**, tyrosine **(16**, Figure S14), isoleucine **(17)**, phenylalanine **(18**, Figure S15), and tryptophan **(21)**, along with three amino acid derivatives; O-acetyl homoserine **(10)**, N-acetyl leucine **(31)**, and N-fructosyl pyroglutamate **(9**, Figure S16). Amino acids have common fragments corresponding to the loss of NH_3_, H_2_O, CO, CO_2_, and CH_2_CO groups (17, 18, 28, 44, and 42 mass units, respectively) [76]. Homoserine **(8)** was the major amino acid identified. It was previously found at high concentration (214.9 mg/g dry extract) in PS flowering shoots and isolated therefrom [77]. Amino acids are essential for human health as they act as precursors for the synthesis of proteins and neurotransmitters, such as serotonin and dopamine, hormones, such as thyroxine, epinephrine, and norepinephrine, and antioxidants, such as glutathione and taurine. They also act as key regulators of body homeostasis, including metabolic processes, transport and storage of nutrients, regulation of gene expression, immune response, growth, repair, and organ development [78].

#### Organic acids

Some plants accumulate certain organic acids in response to abiotic stress conditions such as drought and salinity. Organic acids, particularly succinic **(13)**, galacturonic, and malic acids, increase the plant resilience in case of long-term drought [79]. Gluconic acid **(3**, Figure S17), threonic acid **(5)**, citric acid **(12)**, succinic acid **(13**, Figure S18), 3-hydroxy-3-methylglutaric acid **(14)**, and 2-isopropylmalic acid **(22)** were identified in PSP and PSLS extracts.

#### Fatty acids and their derivatives

Fatty acids are long chain organic compounds (up to 24 carbons) having a carboxylic group at one end. They may be unsaturated or saturated according to the presence or absence of double bonds [80]. Fatty acids and their corresponding derivatives, such as fatty amides, esters, or amines, are produced by various plants to perform several regulatory roles in intracellular and extracellular signaling [81]. Twelve fatty acid were identified in PSP and PSLS extracts, namely tricladic acid A **(29)**, peroxydisebacic acid **(30)**, azelaic acid **(51)**, 9,12,13-trihydroxyoctadeca-10,15-dienoic acid **(67**, Figure S19), undecanedioic acid **(68)**, 5,8,11-trihydroxyoctadec-9-enoic acid **(71**, Figure S20), octadecanedioic acid **(86)**, stearidonic acid **(89)**, 9-hydroxy-10,12-octadecadienoic acid **(90)**, hydroxyhexadecanoic acid **(95)**, linoleic acid **(96)**, hydroxynonadecanoic acid **(97)**, and oleic acid **(98)**, together with lauryldiethanolamine **(87**, Figure S21). Whereas 12-hydroxyoctadec-9-enoic acid **(91)**, chaulmoogric acid **(92)**, 12-hydroxyoctadecanoic acid **(93)**, and petroselinic acid **(94)** were exclusively identified in PSP extract. The identified fatty acids were detected to large extent in both ESI modes and shared characteristic fragments resulting from the loss of water and CO_2_ molecules, 18 and 44 mass units [45].

#### Monoterpenoids

Monoterpenoids constitute a vast group of natural products widely spread in plants. They are part of the defensive oleoresins and essential oils produced by aromatic plants. They play a major role in plant defense, attraction of pollinators, and allelopathy [82]. Structurally, the monoterpenoid skeleton consists of ten carbon atoms derived from two C_5_ isoprene units [83]. Sacranoside A **(27)**, a monoterpene diglycoside, was identified in PSLS extract. Whereas cannabiside D **(38)**, a monoterpene glycoside, and its aglycone **(50)**, together with loliolide **(52**, Figure S22), a monoterpene lactone, were identified in PSP and PSLS extracts. Some monoterpenoids were reported to exhibit anti-inflammatory and anticancer activities [82].

#### Miscellaneous compounds

Other miscellaneous compounds identified in PSP and PSLS extracts included glucose **(6)**, pantothenic acid (vitamin B5) **(19**, Figure S23), triacetin (triglyceride) **(20)**, catechol **(24)**, indoleacrylic acid **(26)**, along with secoisolariciresinol (lignan) **(49)**, pterocarpin (pterocarpan) **(70)**, and soyasaponin Bb (saponin glycoside) **(76)**.

### MN study

The constructed network from the LC-MS/MS data (Fig. 3**)** comprised 155 nodes, including 38 clusters with at least 2 connected nodes, along with 160 self-looped nodes. Clusters of particular interest were clusters A to F, consisting of different types of identified flavonoids such as flavonol and flavone glycosides, flavanones, and isoflavones. Clusters G, H, and I contained some of the identified fatty acids, saponins, and amino acid derivatives, respectively. The self-looped nodes encompassed mainly metabolites belonging to different classes including flavonoids, fatty acids, and some organic acids.

### Network pharmacology study of potential therapeutic activity against breast cancer

#### Compound-target-disease network

Analysis of the compound-target-disease network (Fig. 5; Table 2) revealed that kaempferol-*O*-neohesperidoside **(37)** showed the highest degree of involvement, betweenness, and closeness scores, indicating its interconnection to multiple nodes (protein targets) and suggesting its potential as key active metabolite. Kaempferol-*O*-hexuronide **(33)**, kaempferol-3,7-*O*-di deoxyhexoside **(42)**, and kaempferol-*O*-neohesperidoside **(37)** completed the top of the ranking. On the other hand, carbonic anhydrases, namely CA12, CA2, and CA1, were the major breast cancer protein targets interconnected to the identified metabolites according to their degree of involvement (Table S1). CAs are membrane-associated enzymes overexpressed in several types of cancer cells, including breast, ovarian, renal, and colorectal carcinomas, as well as astrocytic glioma and non-small cell lung cancer. Their tumorigenic effect relies on the regulation of tumor cell intracellular pH and acidification of the extracellular environment. Consequently, this confers tumor cells growth and survival advantages over normal cells, which are unable to adapt to acidity [84, 85].

#### PPI network

PPI network analysis revealed that tumor protein p53 (TP53), serine/threonine kinase 1 (AKT1), and epidermal growth factor receptor (EGFR) were the main hub targets i.e., the most interconnected nodes within the network, based on the degree of nodes sizes TP53 is highly related to breast cancer, as mutations in TP53 are found in around 30% of breast carcinomas, resulting in the loss of its function as a tumor-suppressing protein [86]. Conversely, AKT1 is overactivated in breast cancer, leading to increased mammary tumor growth [87]. Additionally, EGFR is overexpressed in around 14% of breast carcinomas. Overexpression of EGFR induces cell growth, differentiation, angiogenesis, and blocks apoptosis [88].

#### GO and KEGG pathway enrichment analysis

The enrichment analysis suggested that the identified metabolites of PS wastes would inhibit tyrosine kinases which are key enzymes in breast and other types of cancers [89].

In summary, the identified metabolites had the potential to counteract breast cancer by interacting with key enzymes and protein biomarkers involved in the survival and proliferation of breast carcinoma. Therefore, it was crucial to confirm this potential bioactivity by evaluating cytotoxic activity using the *in*-*vitro* MTT assay in MCF-7 and MCF-10a cell lines.

### MTT cytotoxicity assay

Results of the MTT cytotoxicity assay revealed that PSLS extract exhibited a potent cytotoxic activity on MCF-7 cell lines with good selectivity (IC_50 =_ 17.67 and 62.07 µg/mL for MCF-7 and MCF-10a cell lines, respectively, and selectivity index of 3.51), whereas PSP extract showed less potency and less selectivity (IC_50 =_ 32.92 and 53.30 µg/mL for MCF-7 and MCF-10a cell lines, respectively, and selectivity index of 1.62). This could be explained by the higher abundance of phenolic metabolites in PSLS extract compared to PSP as noticed from the LC-MS/MS analysis and MN. As for the isolated components, methyl cis *p*-coumarate showed the strongest cytotoxic activity on MCF-7 cell line followed by trans *p-*coumaric acid then the liquiritigenin/7-methyl liquiritigenin mixture with IC_50_ values of 1.18, 4.49, and 11.10 µg/mL, respectively. Additionally, Methyl cis *p*-coumarate exhibited a significantly higher selectivity index compared to the reference drug doxorubicin, the extracts, and the rest of isolated components, as shown in Table 3.

Regarding the potential mechanisms of action of the isolated components, the subnetwork in Fig. 5B indicates that p-coumaric acid, liquiritigenin, and 7-methyl liquiritigenin interacted with several protein targets associated with breast cancer including CA 1, 2, 4, 9, and 12, as well as aldo-keto reductase family 1 member B1 (AKR1B1), adenosine A3 receptor (ADORA3), protein tyrosine phosphatase non-receptor type 1 (PTPN1), and estrogen receptor 2 (ESR2). Additionally, liquiritigenin and 7-methyl liquiritigenin interacted with cytochrome P19A1 (CYP19A1) and acetyl choline esterase (ACHE), and *p*-coumaric acid with EGFR. This could explain the mechanism of their cytotoxicity against the studied cell lines.

## Conclusion

The metabolite profiles of the 70% ethanolic extracts of PSP and PSLS wastes were established using LC-MS/MS untargeted metabolomics and MN. The results led to the identification of 98 metabolites belonging to different classes including flavonoids and their derivatives, phenolic acids, amino acids and their derivatives, organic acids, fatty acids and their derivatives, monoterpenoids, and other miscellaneous compounds. The identified metabolites were subjected to network pharmacology, which indicated their promising potential for prevention and treatment of breast cancer through the interaction with different enzymes and proteins involved in breast cancer progression. Flavonoids and their derivatives, as well as phenolic acids prevailed, especially in the PSLS extract. These metabolite categories comprised almost half of the total number of identified metabolites and also were the key metabolites interacting with breast cancer targets. As a result, PSLS extract was targeted for the isolation of phenolic components, where trans *p*-coumaric acid, methyl cis *p*-coumarate along with a mixture of liquiritigenin and 7-methyl liquiritigenin, were isolated. The in-vitro cytotoxic activity of the waste extracts and the isolated components were investigated on MCF-7 breast carcinoma and MCF-10a human mammary epithelial cell lines. The results revealed the more potent cytotoxic activity and considerable selectivity of the PSLS extract (IC_50_ = 17.67 ± 1.12 µg/mL and 62.07 ± 5.86 µg/mL on MCF-7 and MCF-10a, respectively) compared to the PSP extract (IC_50_ = 32.92 ± 1.64 µg/mL and 53.30 ± 3.48 µg/mL on MCF-7 and MCF-10a, respectively). This could be attributed to the higher abundance in PSLS extract of metabolites belonging to phenolic categories (flavonoids and phenolic acids). The isolated PSLS components also showed a potent cytotoxic activity with high selectivity index compared to the reference drug doxorubicin. Methyl cis *p*-coumarate showed the strongest activity as well as the highest selectivity among the isolated components with IC_50_ values of 1.18 ± 0.05 and 32.36 ± 2.09 µg/mL, which correspond to 6.91 ± 2.73 and 181.97 ± 21.23 µM, on MCF-7 and MCF-10a, respectively. This could be ascribed to their interaction with various breast cancer-associated protein targets such as CAs, AKR1B1, ADORA3, PTPN1, ESR2, and EGFR. These results suggest the possibility for valorizing PS wastes, especially PSLS extract, as potential breast cancer therapeutic agents and as a source of several bioactive phenolic compounds. However, the efficiency and safety of the investigated extracts and isolated components require further confirmation through in-vivo studies and clinical trials.

## Electronic supplementary material

Below is the link to the electronic supplementary material.

Supplementary Material 1

Supplementary Material 2

## Data Availability

The data that support the findings of this study will be made available on reasonable request.

## Declarations

### Clinical trials and informed consents

No human subjects were involved in this article. Informed consents and clinical trial number are not applicable.

### Supporting information

MS/MS spectra of representative members from each metabolite class, schematic representation of extraction, fractionation, and component isolation steps, structures, NMR charts of the isolated components, along with figures of MTT cytotoxicity assay results are attached in a supplementary file.

## Abbreviations

LC-ESI-QTOF-MS/MS: Liquid chromatography-electrospray ionization-quadrupole time-of-flight tandem mass spectrometry
PS: Pisum sativum
LC-MS: Liquid chromatography-mass spectrometry
LC-MS/MS: Liquid chromatography-tandem mass spectrometry
PSP: Pisum sativum peels
PSLS: Pisum sativum leaves and stems
GNPS-MN: Global Natural Products Social Molecular Networking
MN: Molecular networking
TLC: Thin layer chromatography
NMR: Nuclear magnetic resonance
SEA: SwissTargetPrediction database
PPI: Protein protein interaction
GO: Gene ontology
KEGG: Kyoto encyclopedia of genes and genome
MTT: 3-(4,5- dimethylthiazol-2-y1)-2,5-diphenyl tetrazolium bromide
IC_50_: The half-maximal inhibitory concentration
CA: Carbonic anhydrase
TP53: Tumor protein p53
AKT1: Serine/threonine kinase 1
AKR1B1: Aldo-keto reductase family 1 member B1
ADORA3: Adenosine A3 receptor
PTPN1: Protein tyrosine phosphatase non-receptor type 1
ESR2: Estrogen receptor 2
CYP19A1: Cytochrome P19A1
ACHE: Acetyl choline esterase
