# Supplementary figures and images for: Uncovering the therapeutic potential of green pea waste in breast cancer: a multi-target approach utilizing LC-MS/MS metabolomics, molecular networking, and network pharmacology
Source: BMC Complement Med Ther. 2024 Oct 31;24:379. doi: 10.1186/s12906-024-04669-x (PMC11526710; doi:10.1186/s12906-024-04669-x)

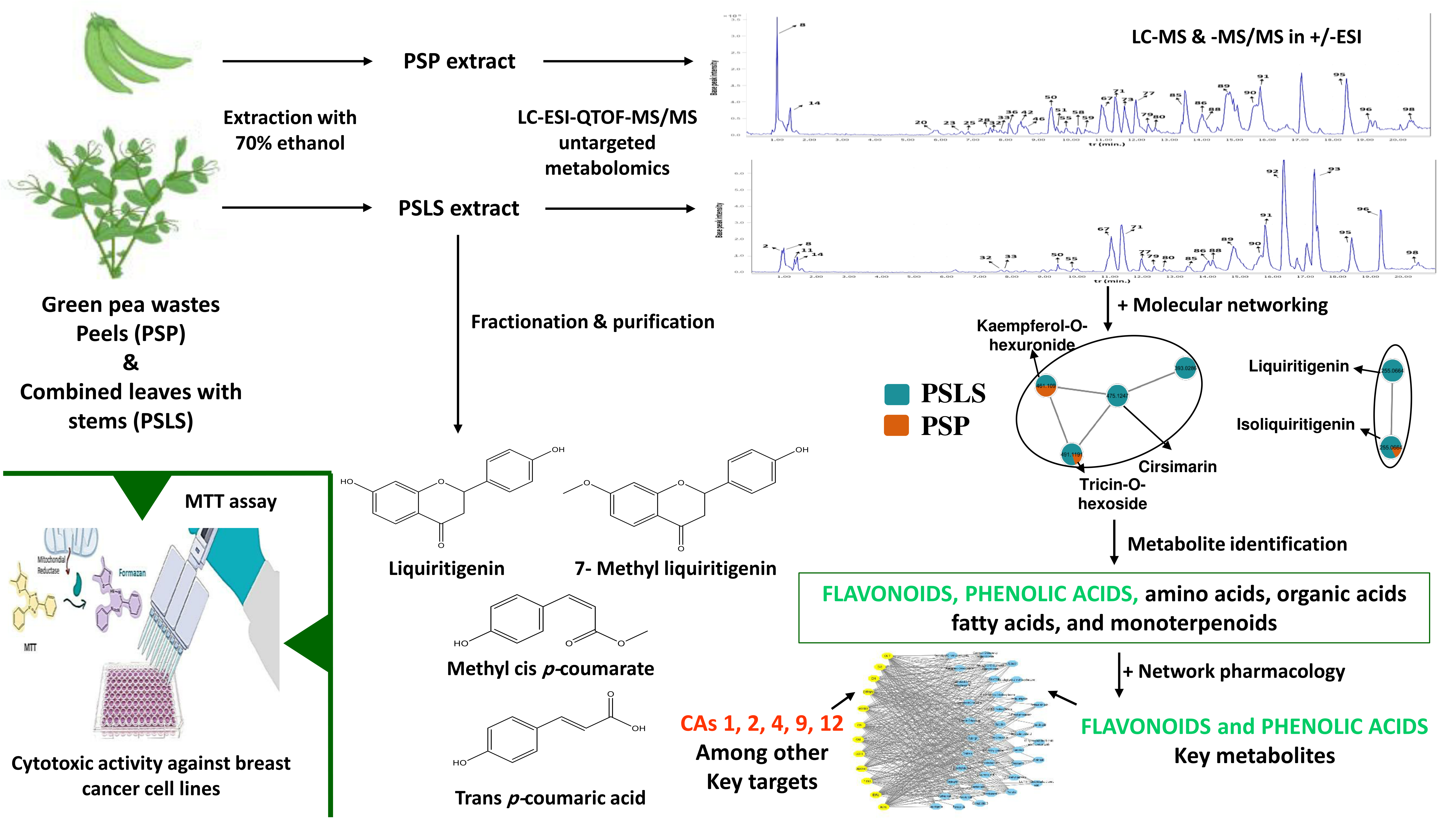

Supplement: Supplementary file 1 — Supplementary Material 1 [file 12906_2024_4669_MOESM1_ESM.tif]
