# Supplementary material for: Uncovering the therapeutic potential of green pea waste in breast cancer: a multi-target approach utilizing LC-MS/MS metabolomics, molecular networking, and network pharmacology
Source: BMC Complement Med Ther. 2024 Oct 31;24:379. doi: 10.1186/s12906-024-04669-x (PMC11526710; doi:10.1186/s12906-024-04669-x)
Supplement: Supplementary file 2 — Supplementary Material 2 [file 12906_2024_4669_MOESM2_ESM.docx]

**Uncovering the therapeutic potential of green pea waste in breast cancer: A multi-target approach utilizing LC-MS/MS metabolomics, molecular networking, and network pharmacology**

Asmaa M. Khalil^1*^, Omar M. Sabry^1,2^, Hesham I. El-Askary^1^, Soheir M. El Zalabani^1^, Basma M. Eltanany^3^, Laura Pont ^4,5^, Fernando Benavente^4^, Ahmed F. Mohamed^6,7^, Nesrin M. Fayek^1^

^1^ Department of Pharmacognosy, Faculty of Pharmacy, Cairo University, 11562 Cairo, Egypt

^2^ Department of Pharmacognosy, Faculty of Pharmacy, Heliopolis University, 4645241 Cairo, Egypt

^3^ Department of Pharmaceutical Analytical Chemistry, Faculty of Pharmacy, Cairo University, 11562 Cairo, Egypt

^4^ Department of Chemical Engineering and Analytical Chemistry, Institute for Research on Nutrition and Food Safety (INSA·UB), University of Barcelona, 08028 Barcelona, Spain

^5^ Serra Húnter Program, Generalitat de Catalunya, 08007 Barcelona, Spain

^6^ Department of Pharmacology and Toxicology, Faculty of Pharmacy, Cairo University, 11562 Cairo, Egypt

^7^ Faculty of Pharmacy, King Salman International University (KSIU), 46612 Ras Sedr, South Sinai, Egypt

* Corresponding author: Asmaa Khalil

E-mail address of the corresponding author:  [asmaa.khalil@pharma.cu.edu.eg](mailto:asmaa.khalil@pharma.cu.edu.eg); [asmaa.khalil@cu.edu.eg](mailto:asmaa.khalil@cu.edu.eg)

**
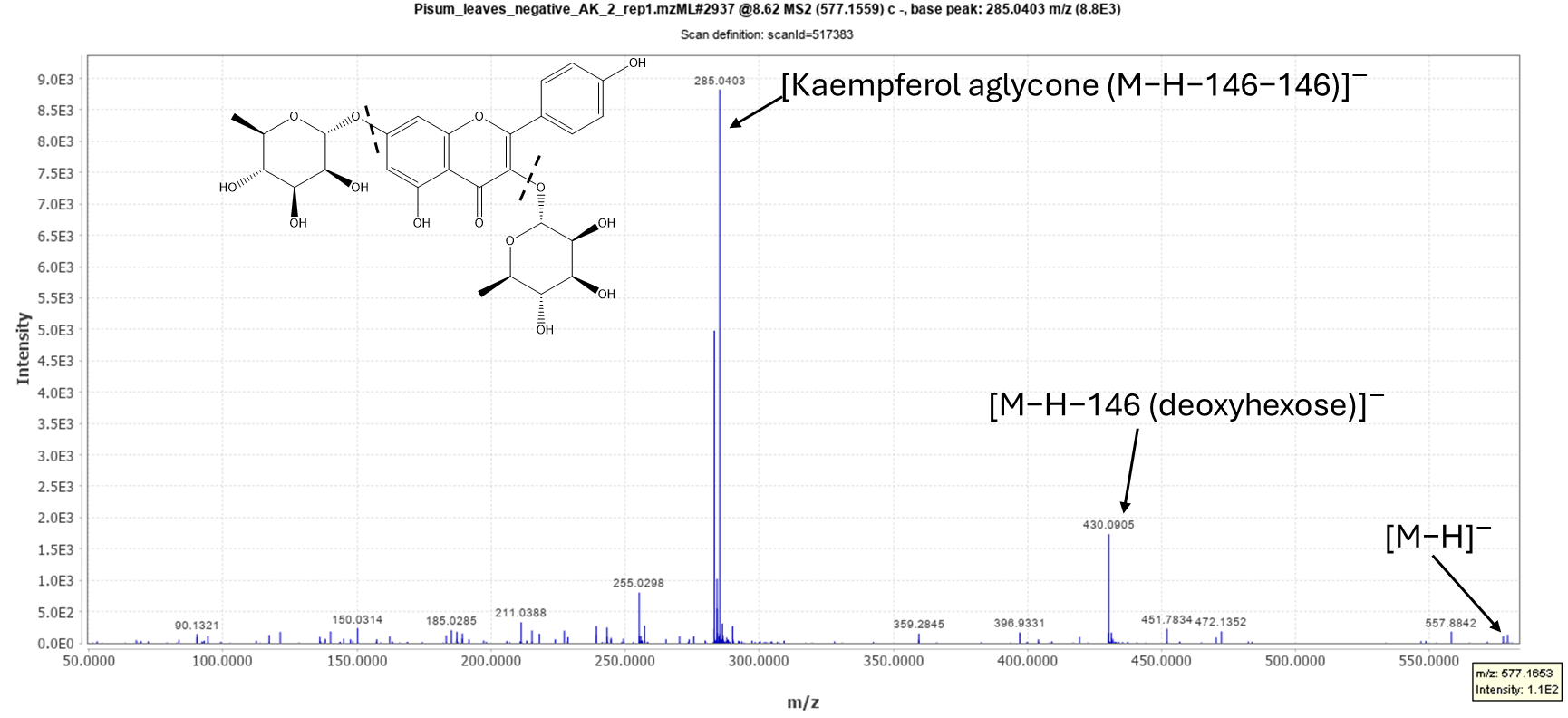
**

**Figure S1.** MS/MS spectrum of kaempferol-3,7-*O*-di deoxyhexoside **(42, Table 1, Table 2)** in negative ESI mode

**
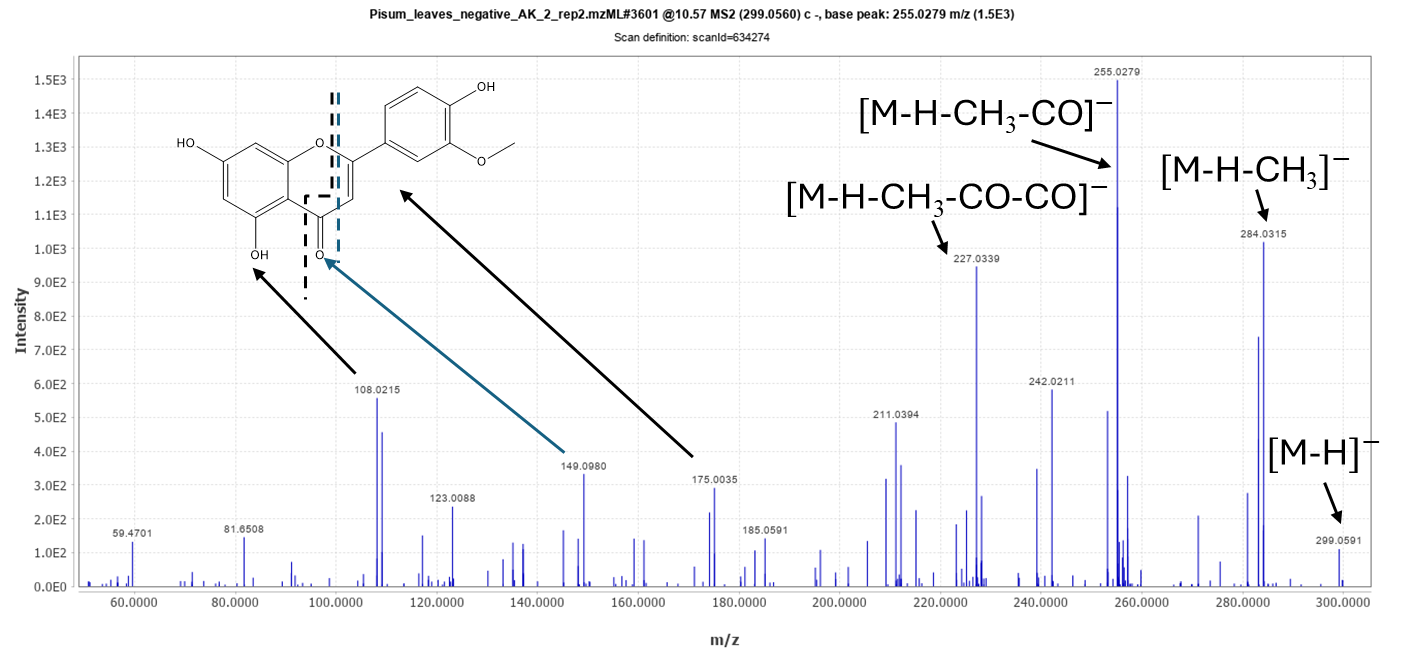
**

**Figure S2.** MS/MS spectrum of chrysoeriol **(62, Table 1)** in the negative ESI mode


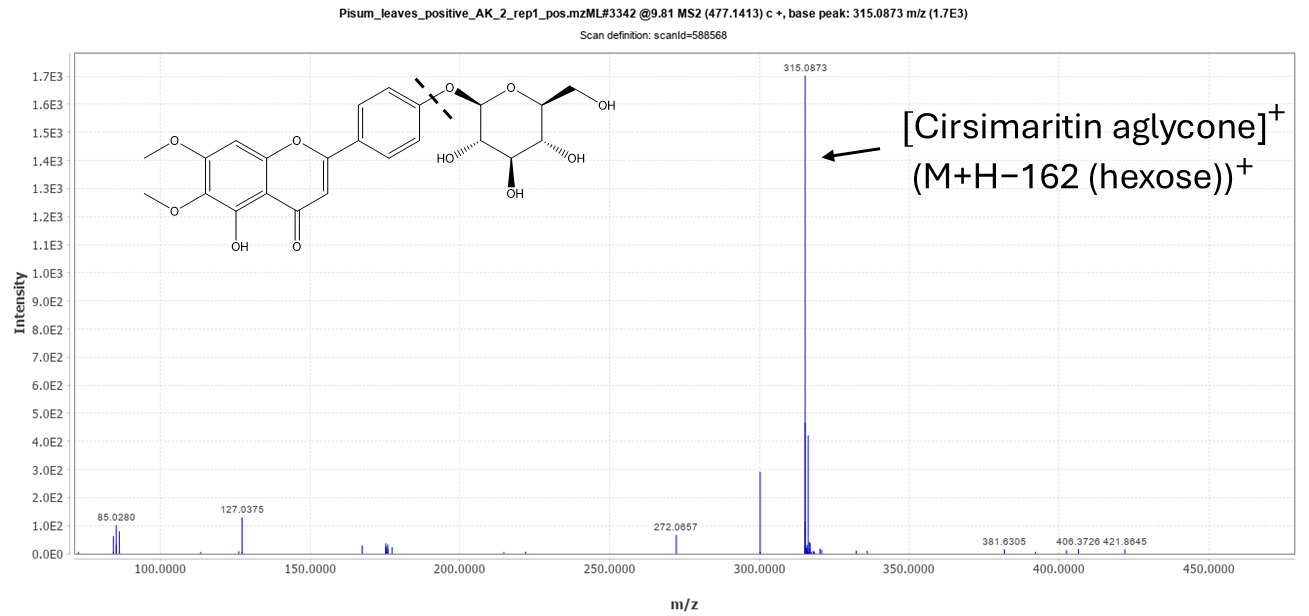


**Figure S3.** MS/MS spectrum of 4 cirsimarin **(54, Table 1)** in positive ESI mode

**
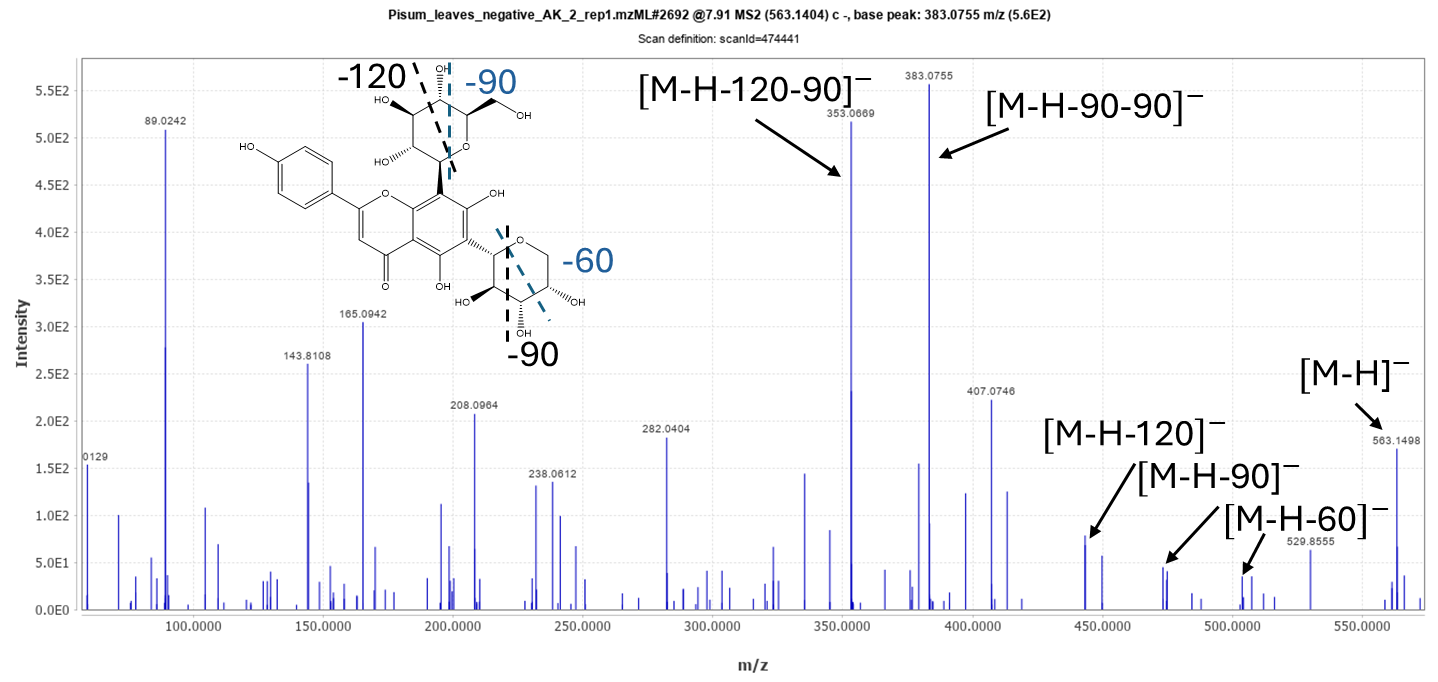
**

**Figure S4.** MS/MS spectrum of apigenin-6-*C*-pentoside-8-*C*-hexoside **(34, Table 1)** in negative ESI mode


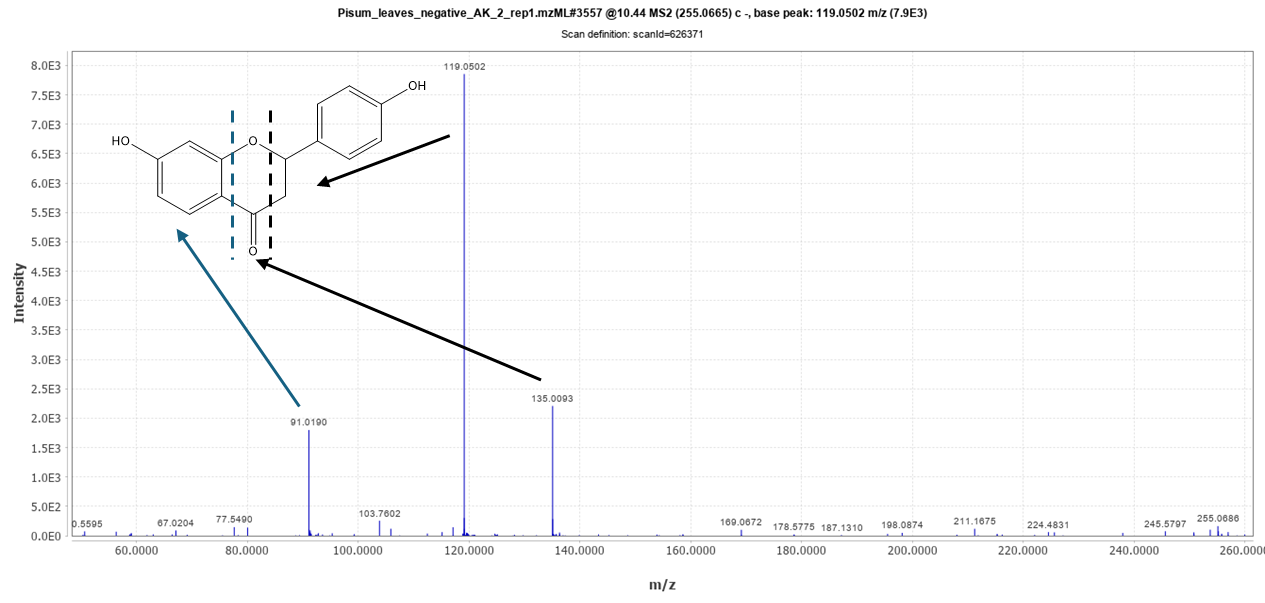


**Figure S5.** MS/MS spectrum of liquiritigenin **(58, Table 1, Table 2)** in negative ESI mode


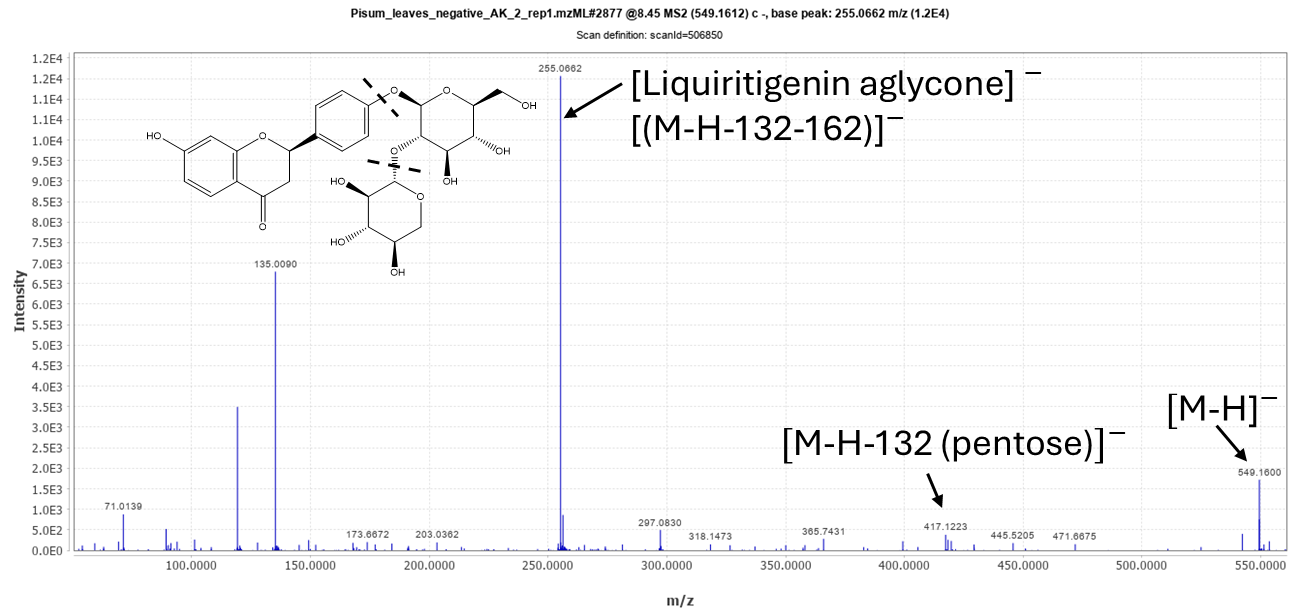


**Figure S6.** MS/MS spectrum of liquiritigenin-O-pentosyl hexoside **(39, Table 1)** in negative ESI mode


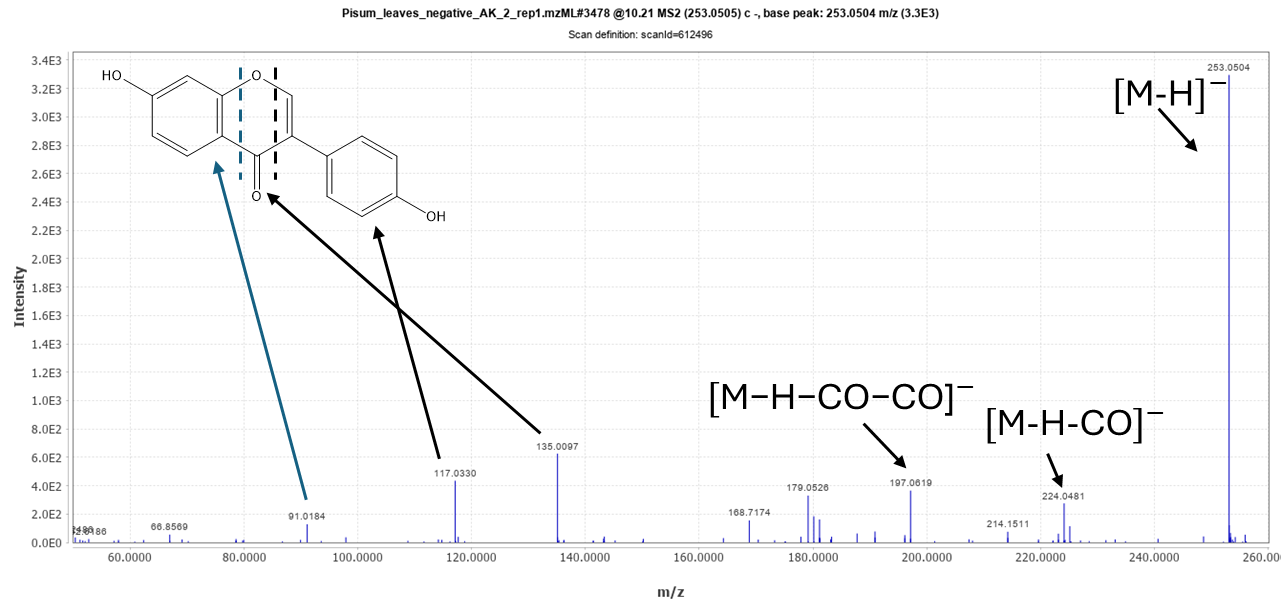


**Figure S7.** MS/MS spectrum of daidzein **(57, Table 1, Table 2)** in negative ESI mode


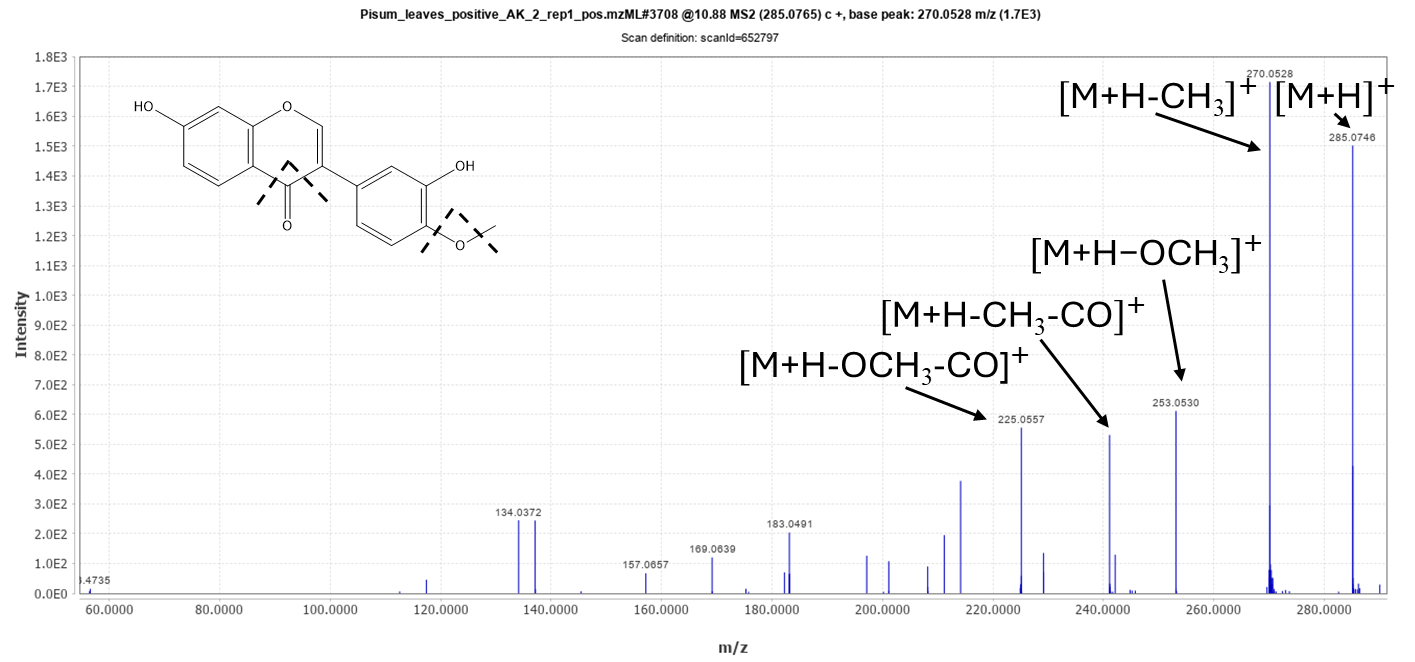


**Figure S8.** MS/MS spectrum of calycosin **(64, Table 1, Table 2)** in positive ESI mode


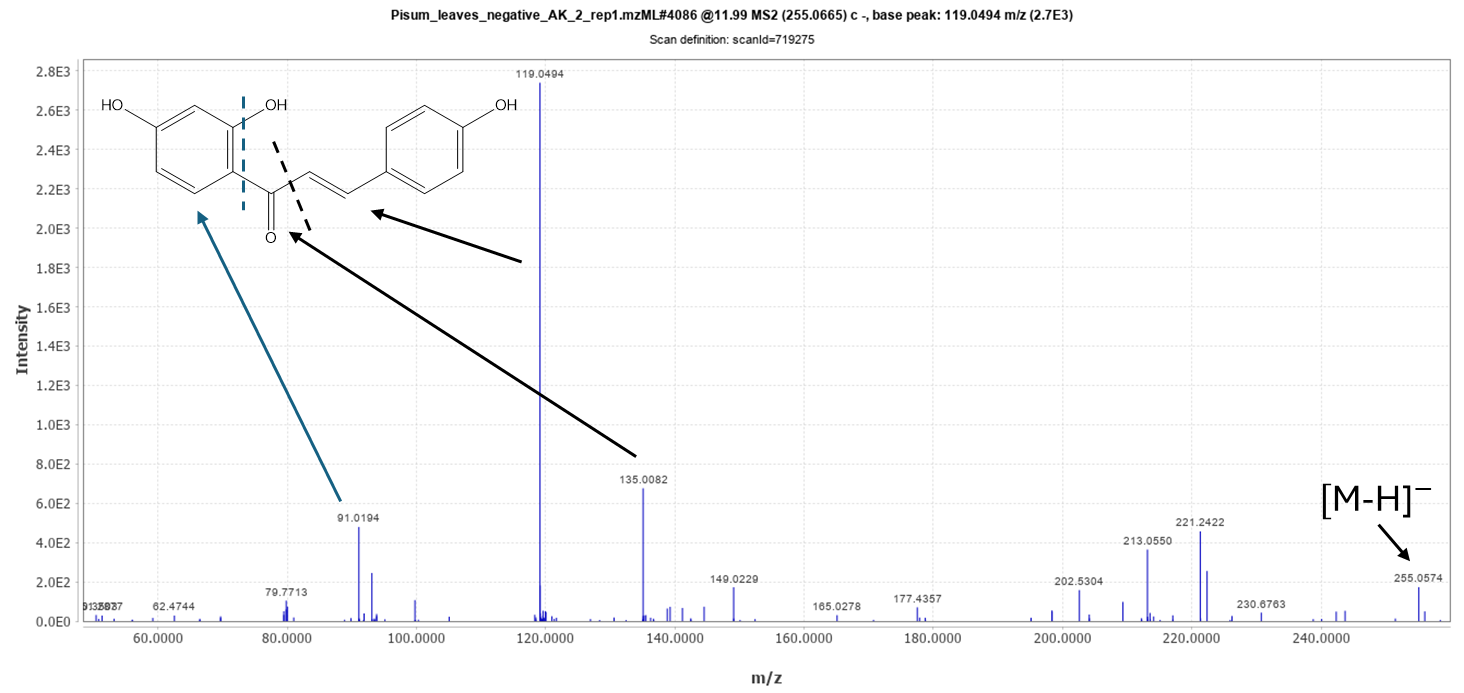


**Figure S9.** MS/MS spectrum of isoliquiritigenin **(77, Table 1, Table 2)** in negative ESI mode


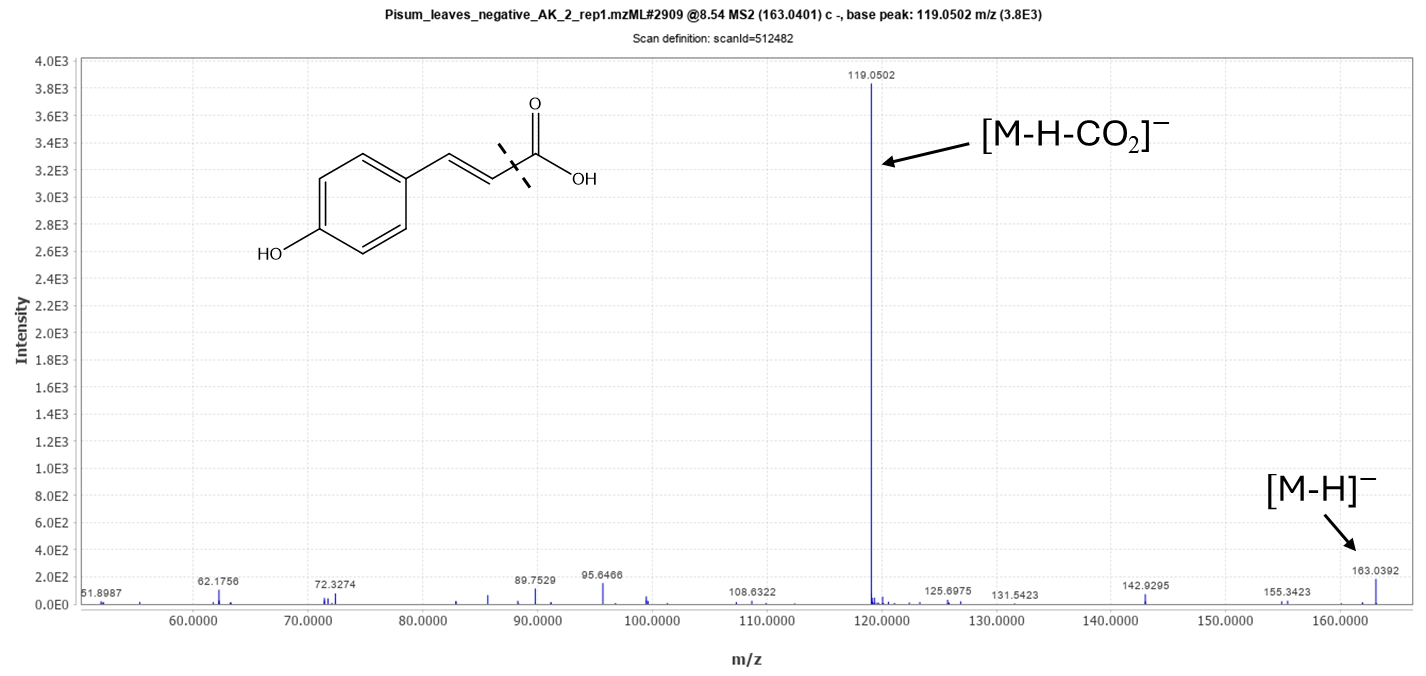


**Figure S10.** MS/MS spectrum of *p*-coumaric acid **(40, Table 1, Table 2)** in negative ESI mode


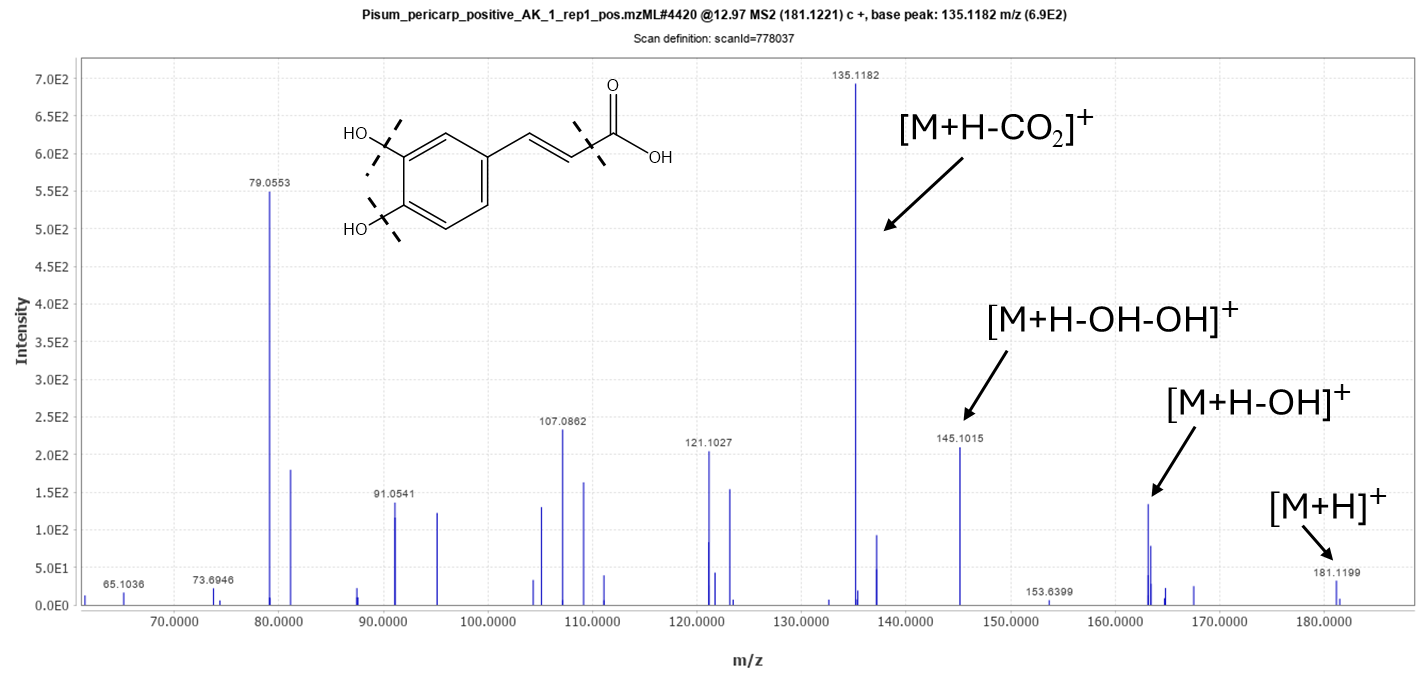


**Figure S11.** MS/MS Spectrum of caffeic acid **(83, Table 1, Table 2)** in positive ESI mode


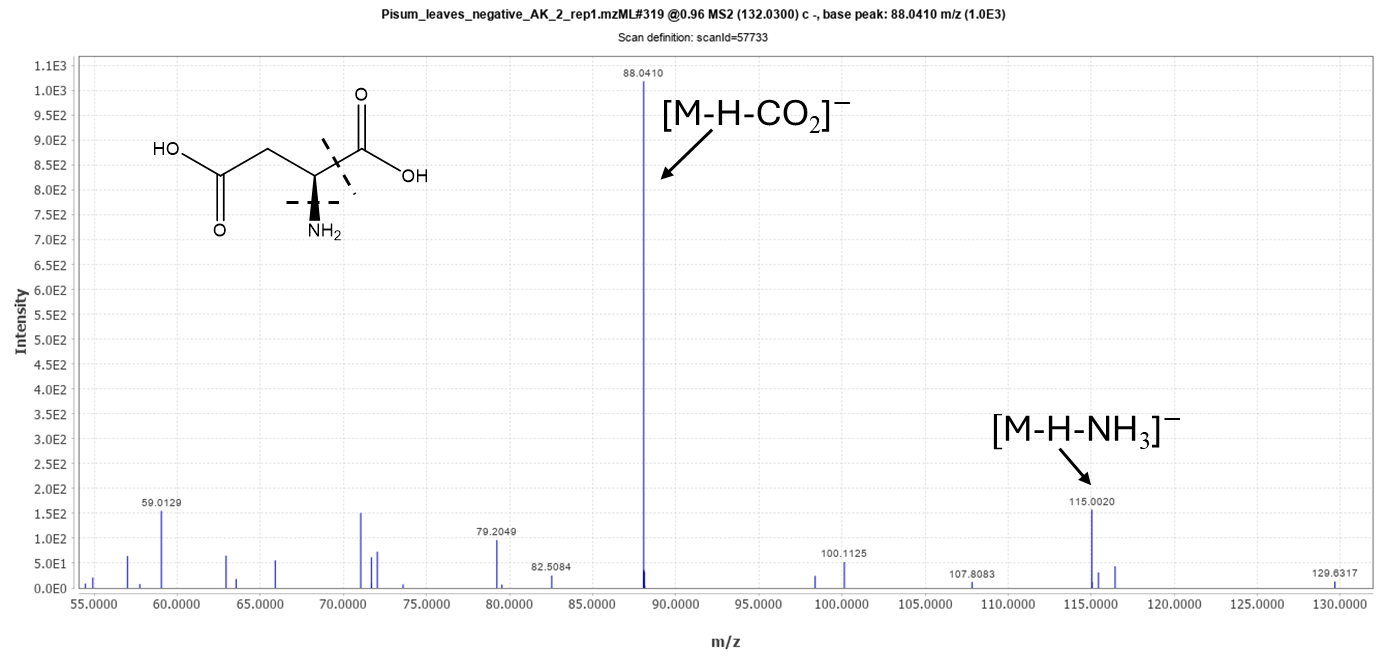


**Figure S12.** MS/MS spectrum of aspartic acid **(2, Table 1)** in negative ESI mode


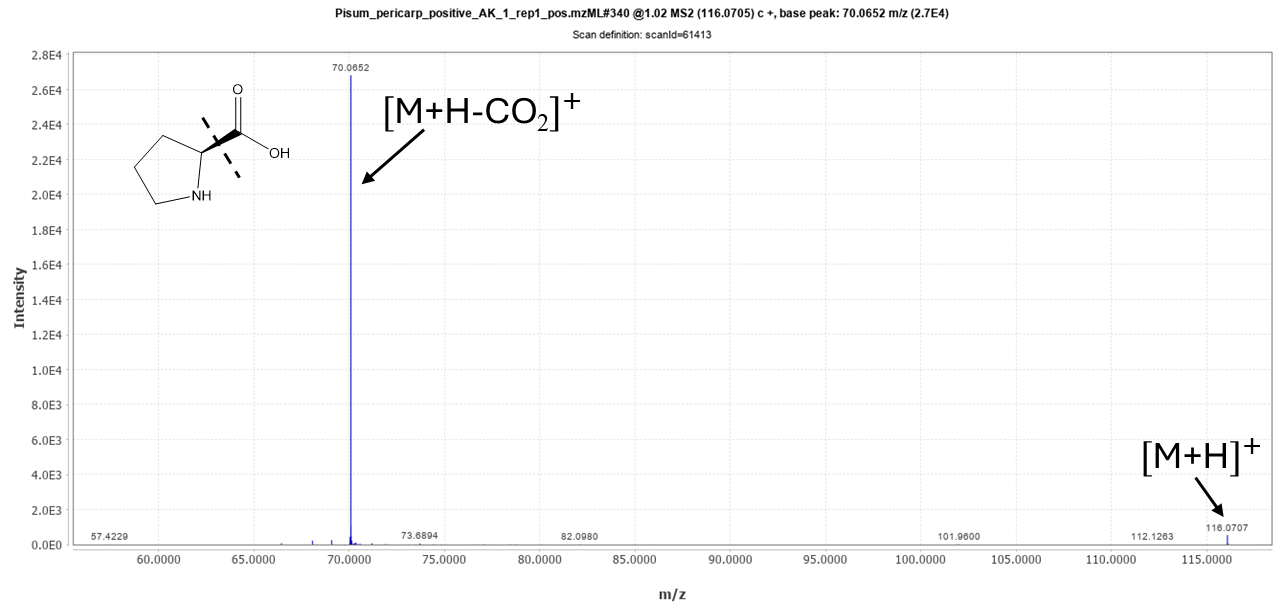


**Figure S13.** MS/MS spectrum of proline **(7, Table 1)** in positive ESI mode

**
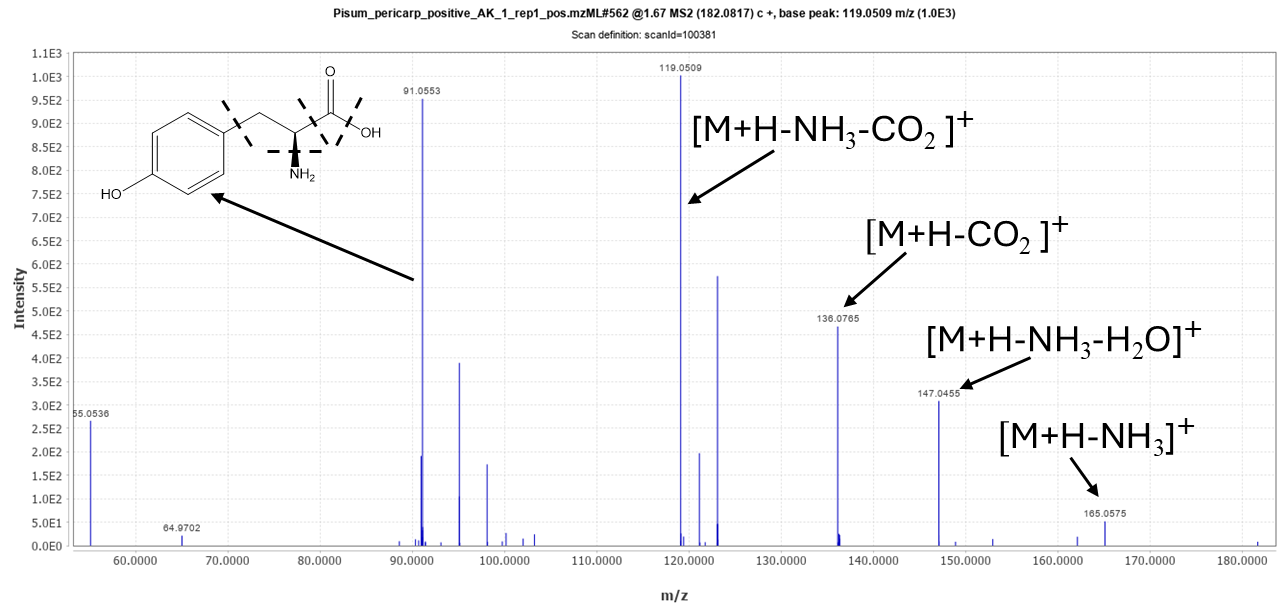
**

**Figure S14.** MS/MS spectrum of tyrosine **(16, Table 1)** in positive ESI mode


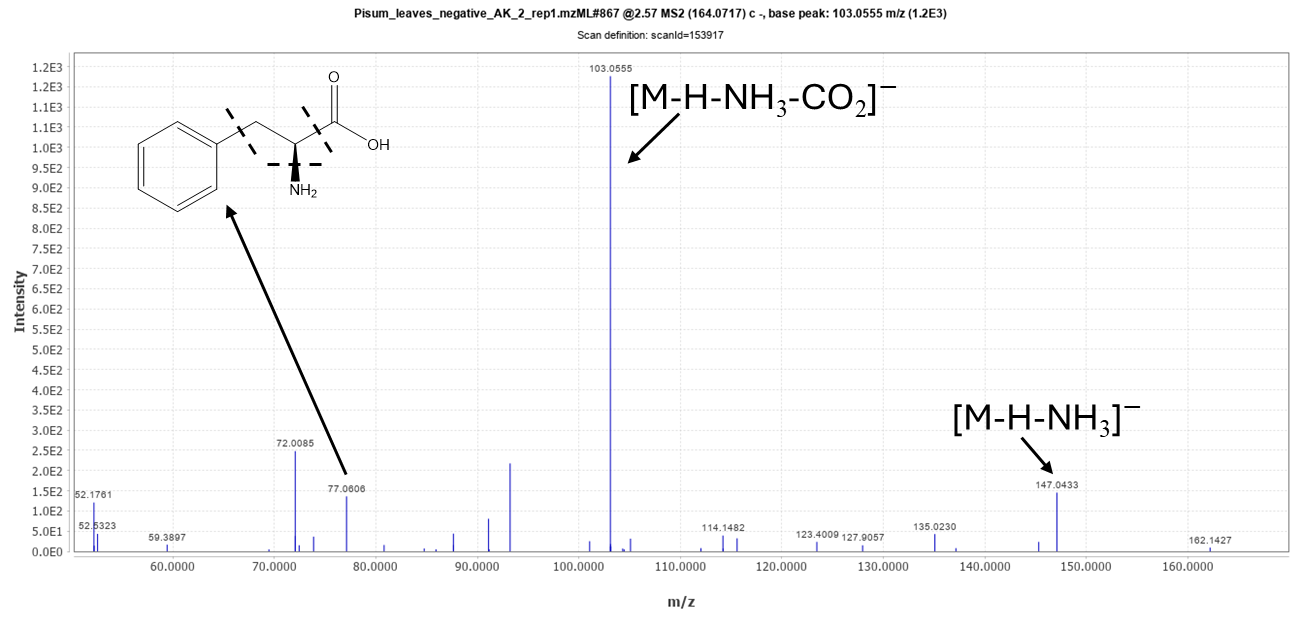


**Figure S15.** MS/MS spectrum of phenylalanine **(18, Table 1)** in negative ESI mode

**
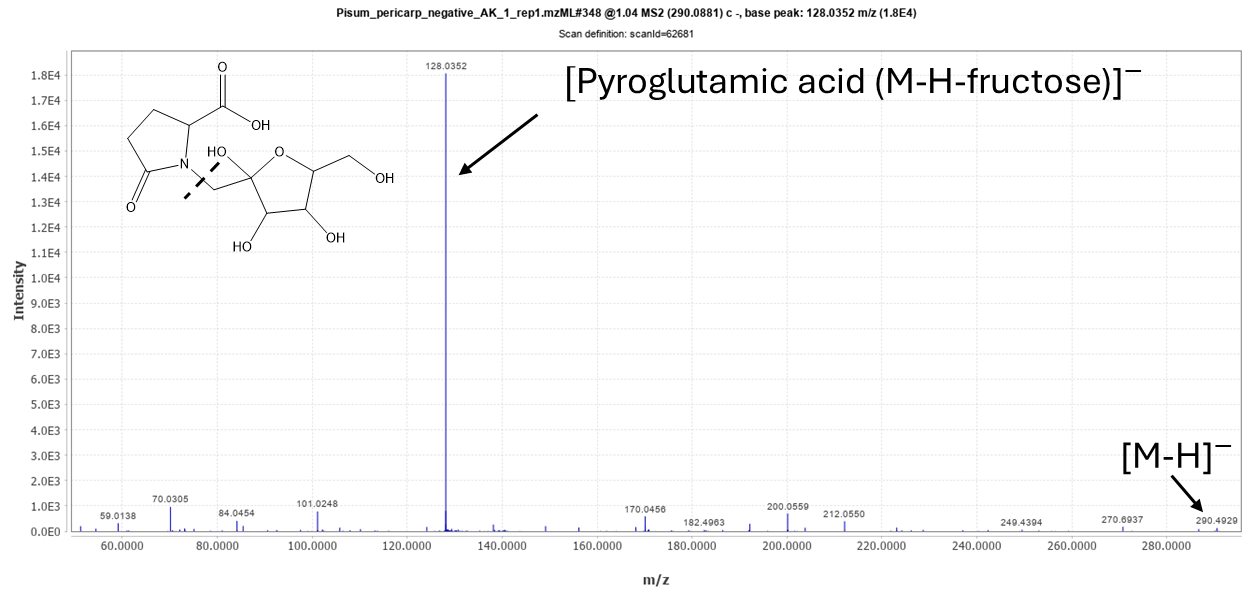
**

**Figure S16.** MS/MS spectrum of N-fructosyl pyroglutamate **(9, Table 1)** in negative ESI mode

**
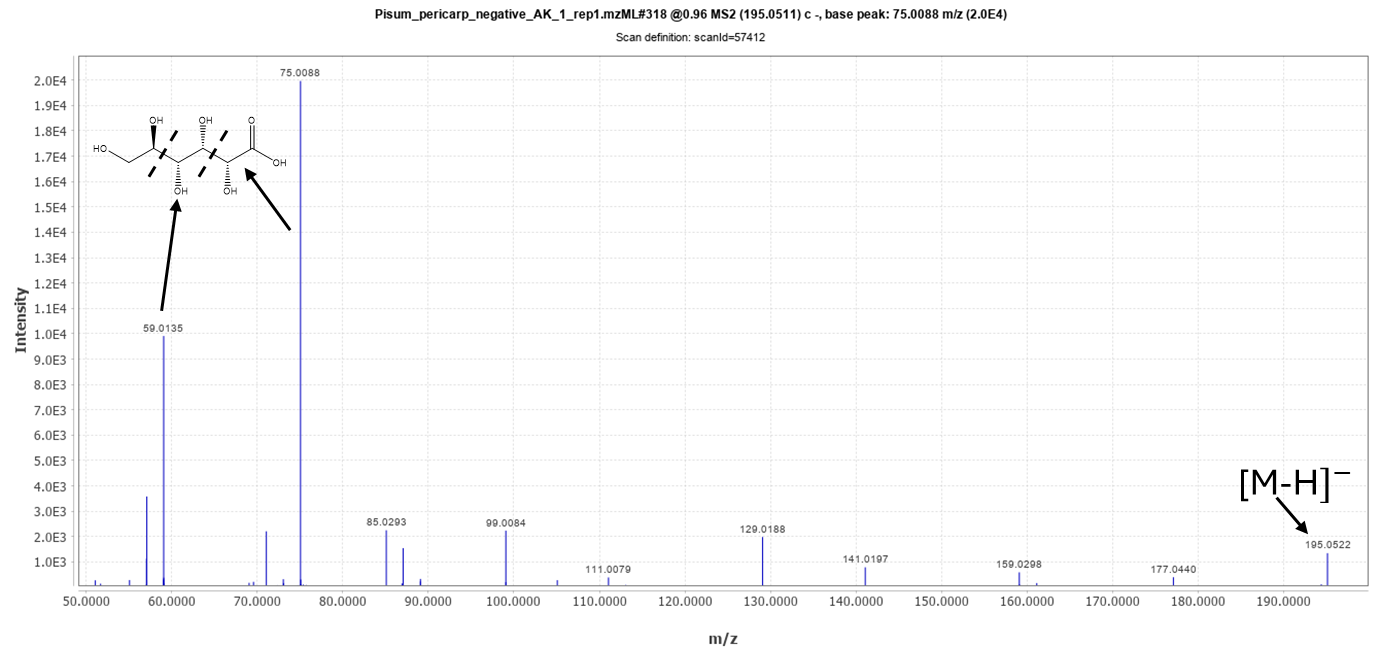
**

**Figure S17.** MS/MS spectrum of gluconic acid **(3, Table 1)** in negative ESI mode

**
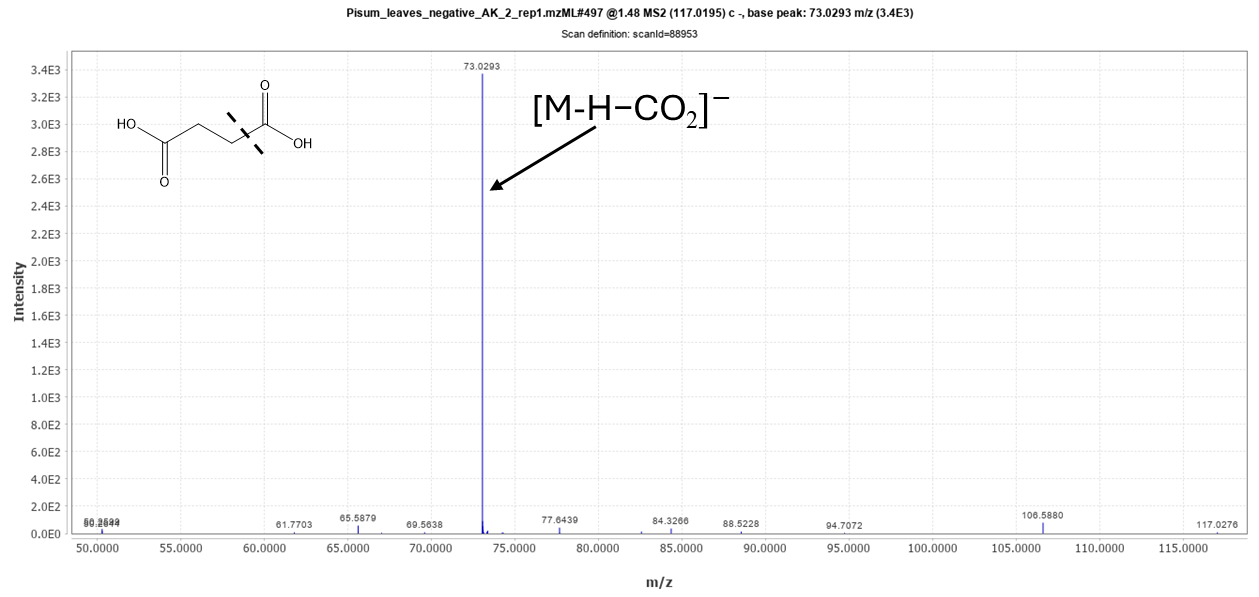
**

**Figure S18.** MS/MS spectrum of succinic acid **(13, Table 1)** in negative ESI mode


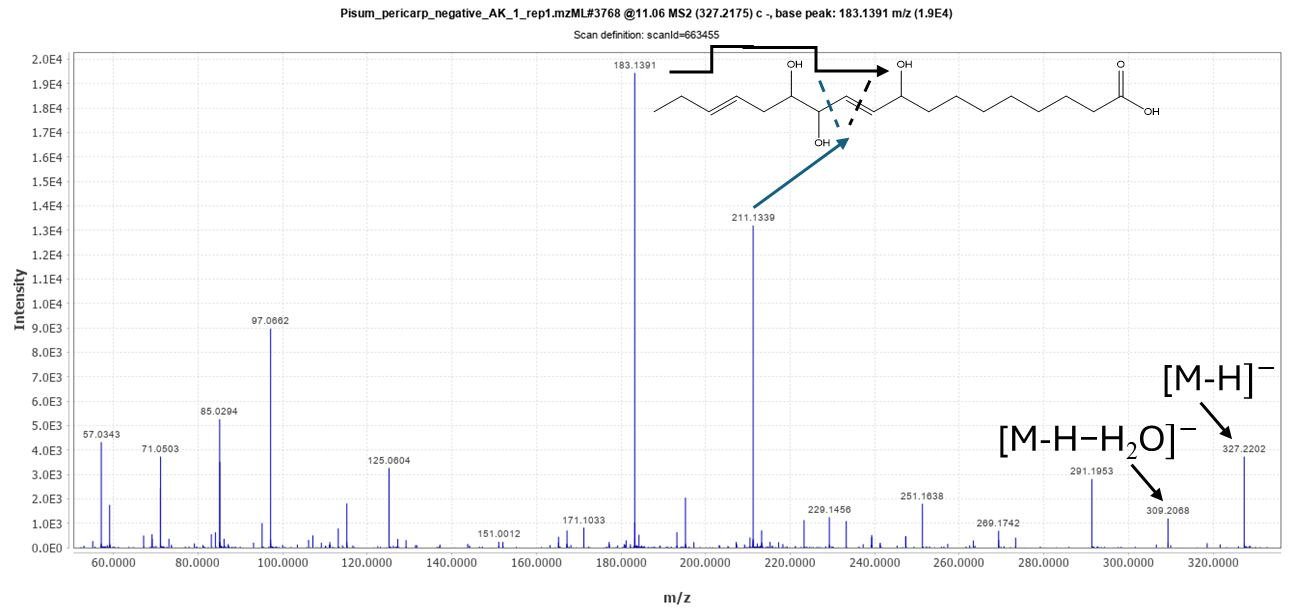


**Figure S19.** MS/MS spectrum of 9,12,13-trihydroxyoctadeca-10,15-dienoic acid **(67, Table 1)** in negative ESI mode


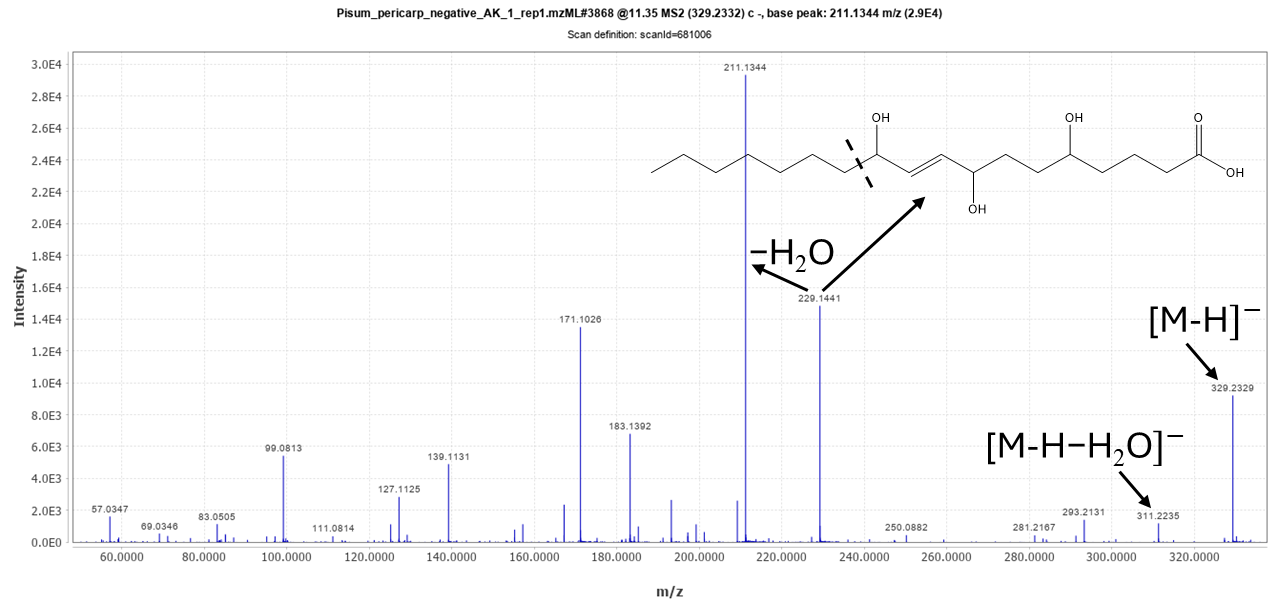


**Figure S20.** MS/MS spectrum of 5,8,11-trihydroxyoctadec-9-enoic acid **(71, Table 1, Table 2)** in negative ESI mode


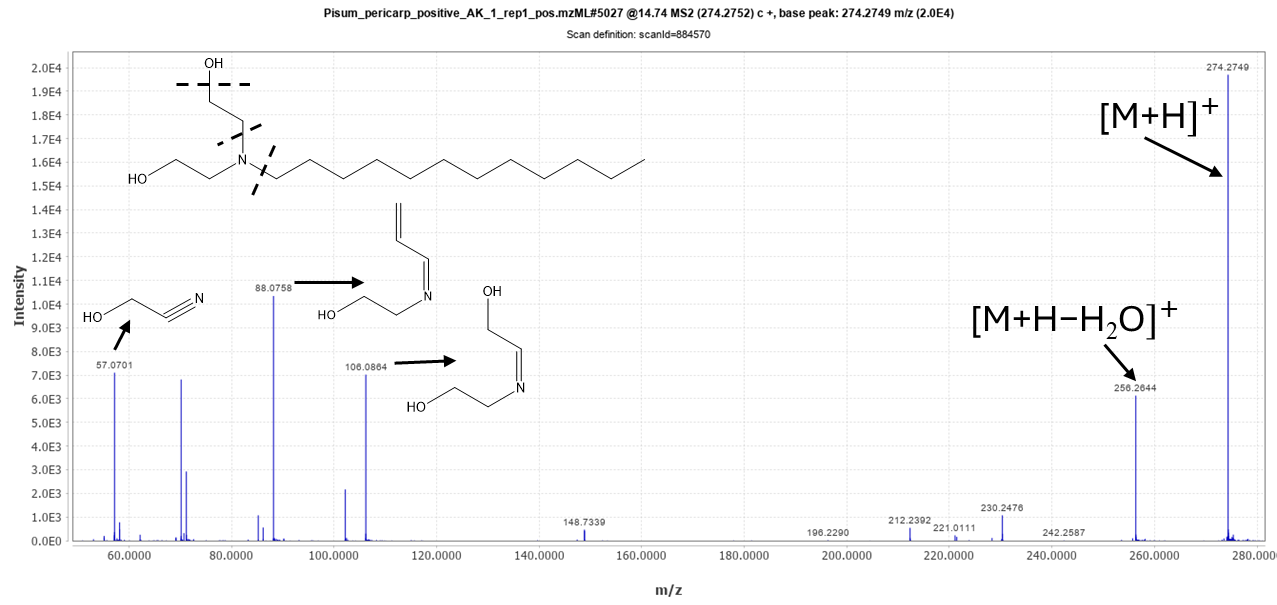


**Figure S21.** MS/MS spectrum of lauryldiethanolamine **(87, Table 1)** in positive ESI mode


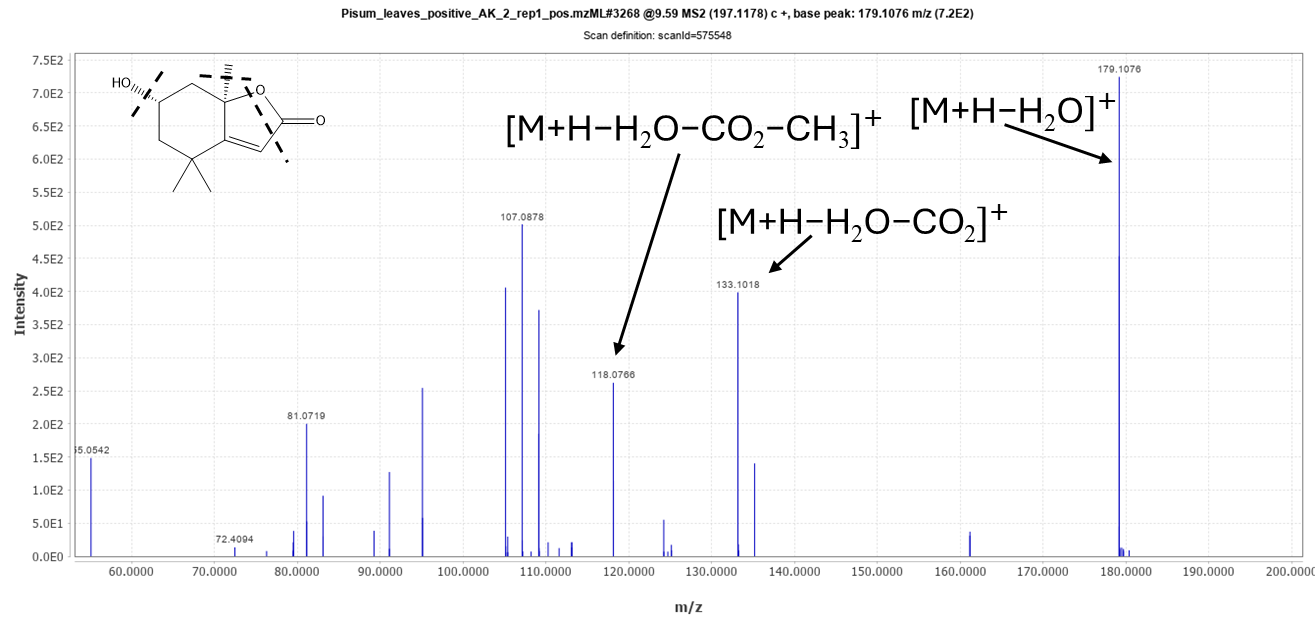


**Figure S22. MS/MS** spectrum of loliolide **(52, Table 1)** in negative ESI mode


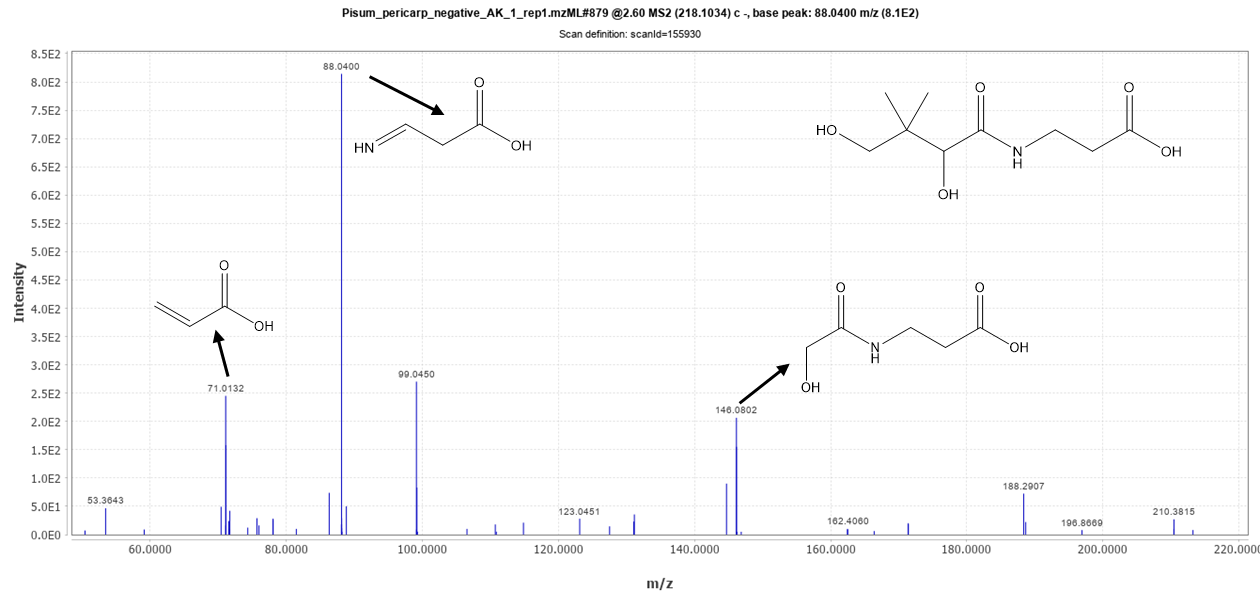


**Figure S23.** MS/MS spectrum of pantothenic acid **(19, Table 1)** in negative ESI mode


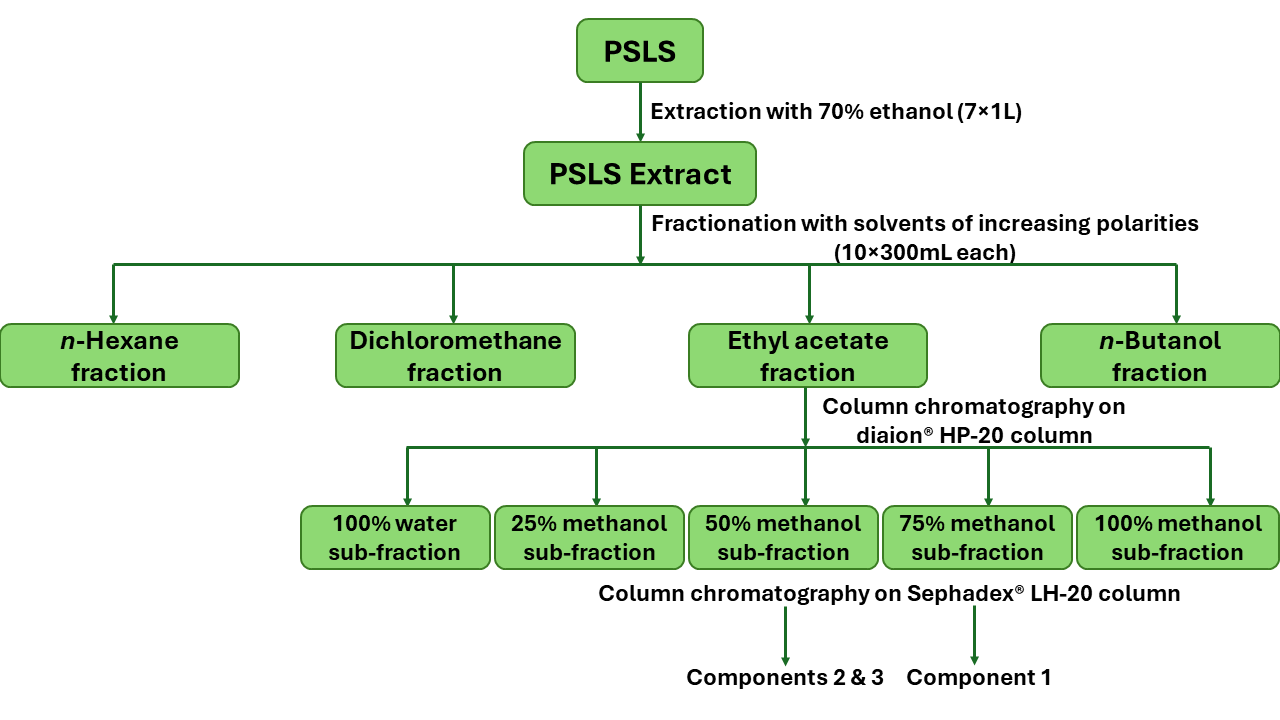


**Figure S24.** Schematic representation of extraction, fractionation, and components isolation steps

**
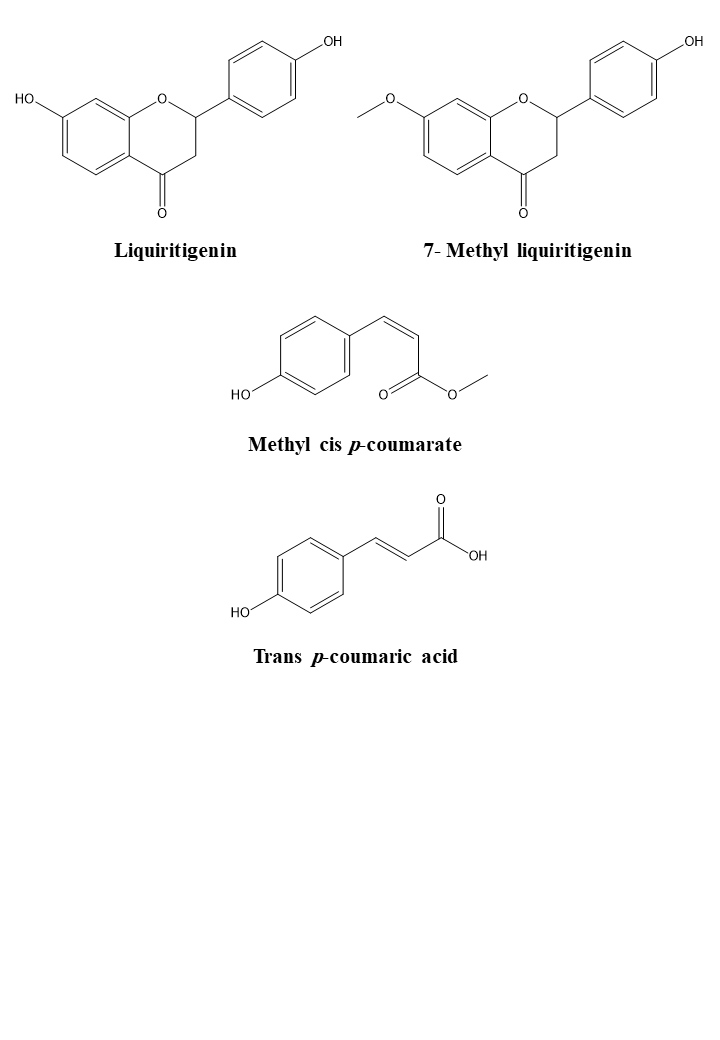
**

**Figure S25.** Structures of the phenolic components isolated from *Pisum sativum* leaves and stems extract (PSLS)


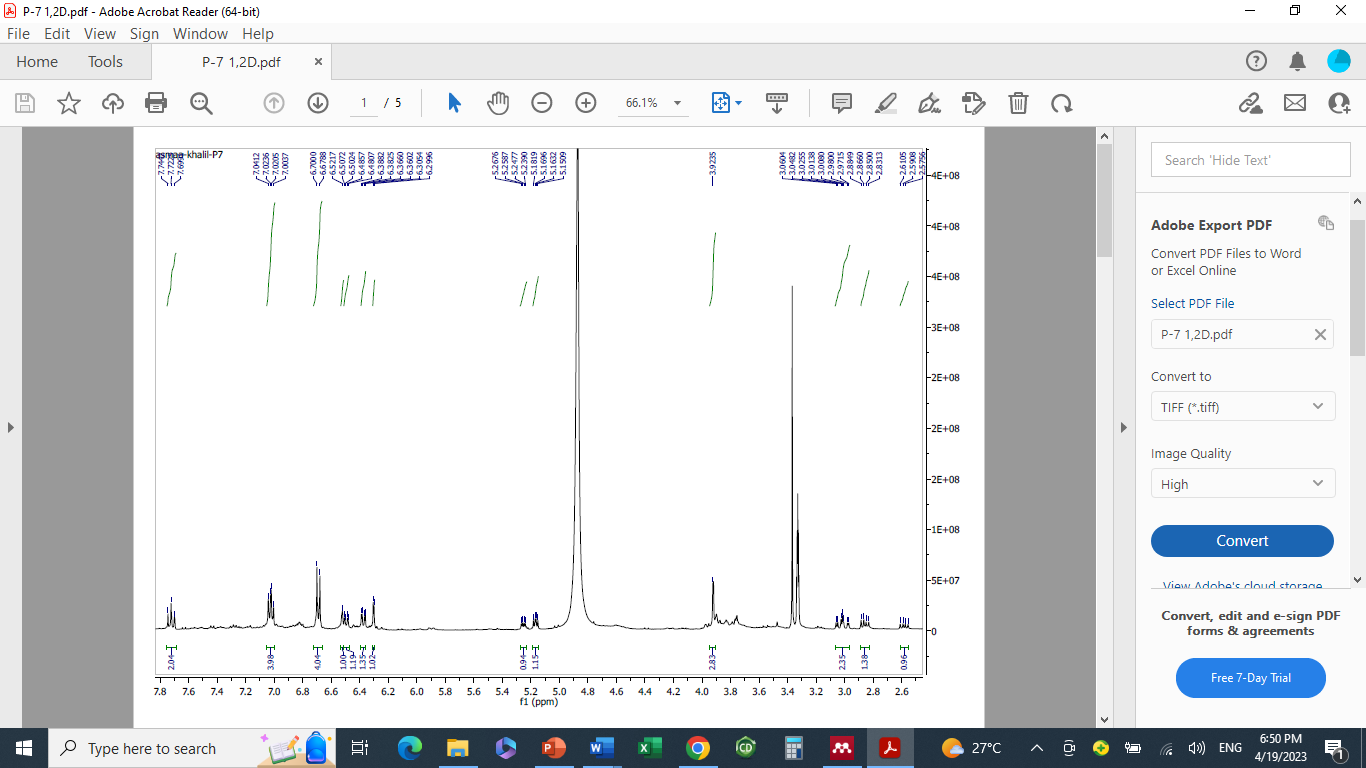


**Figure S26.** H^1^ NMR spectrum of liquiritigenin/7-methyl liquiritigenin mixture, component 1


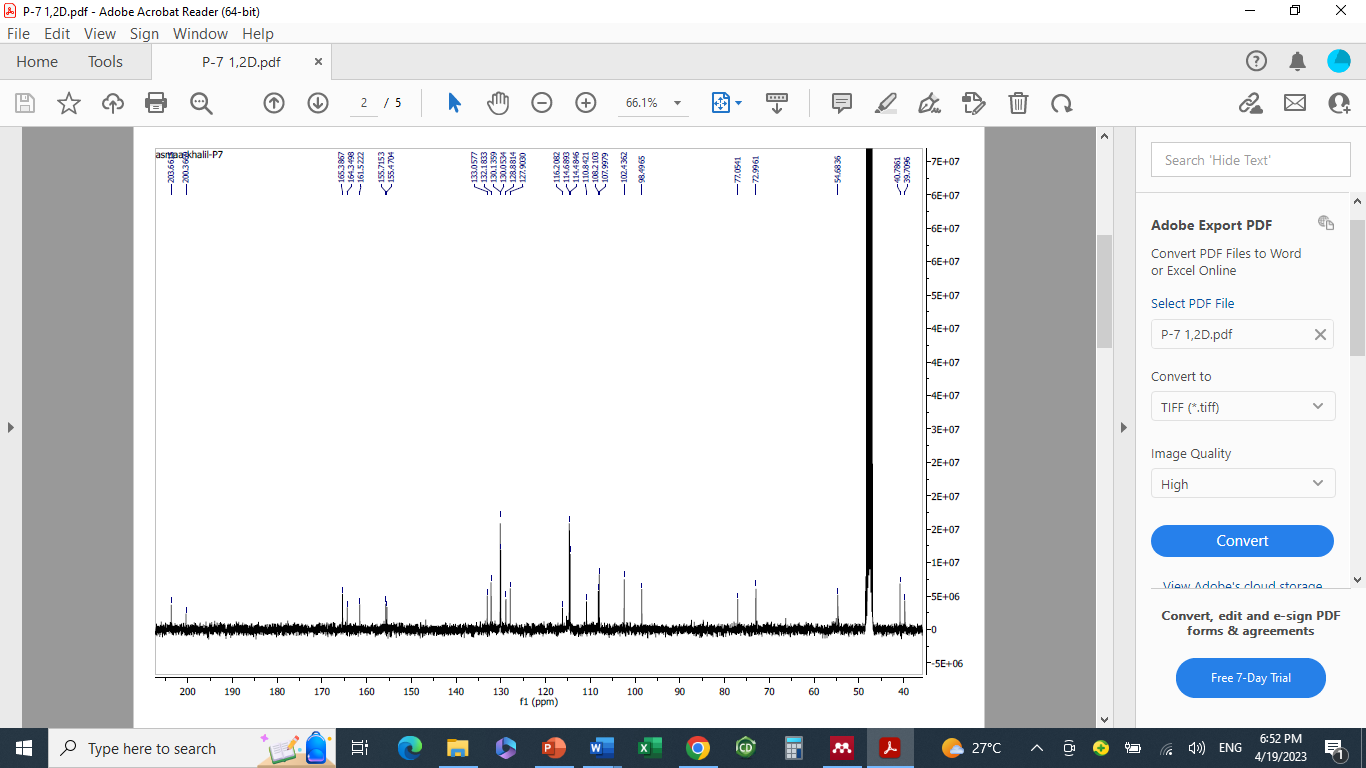


**Figure S27.** C^13^ NMR spectrum of liquiritigenin/7-methyl liquiritigenin mixture, component 1


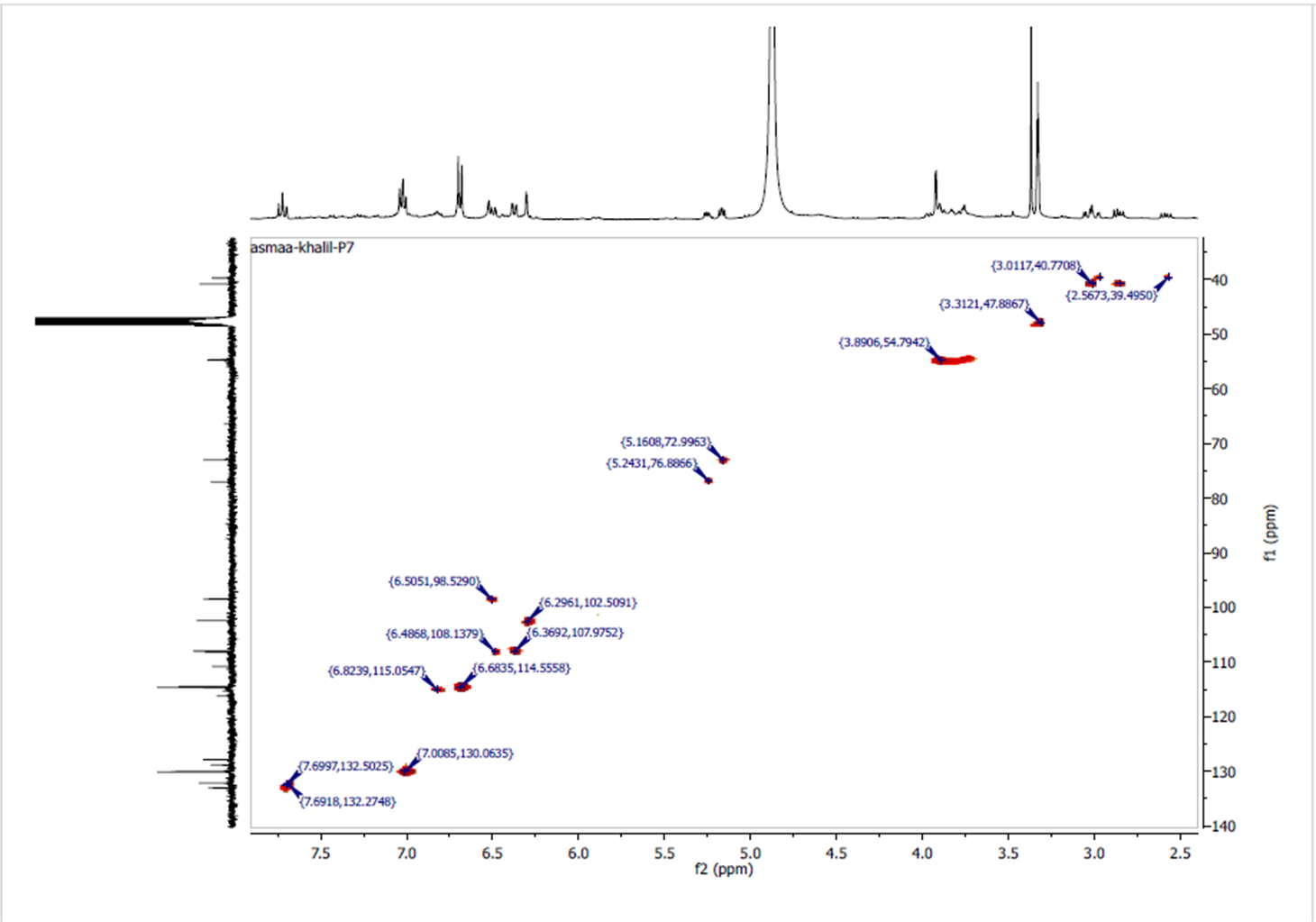


**Figure S28.** HSQC spectrum of liquiritigenin/7-methyl liquiritigenin mixture, component 1


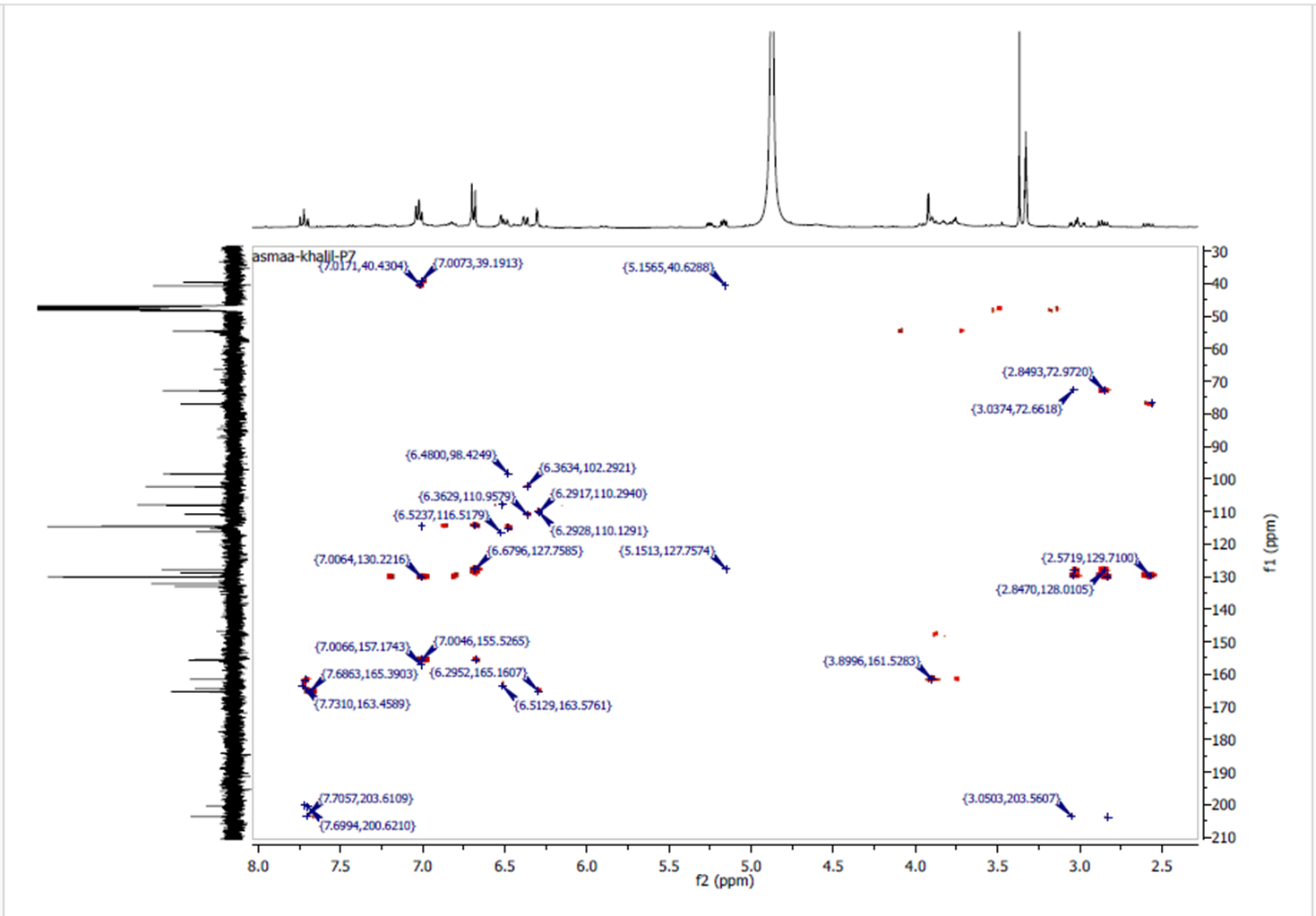


**Figure S29.** HMBC spectrum of liquiritigenin/7-methyl liquiritigenin mixture, component 1


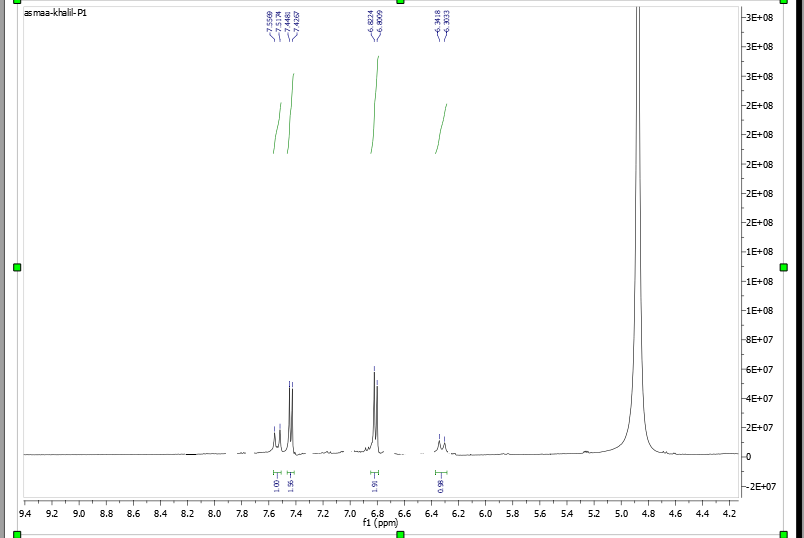

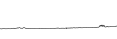

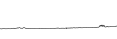

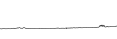


**Figure S30.** H^1^ NMR spectrum of trans *p*-coumaric acid, component 2


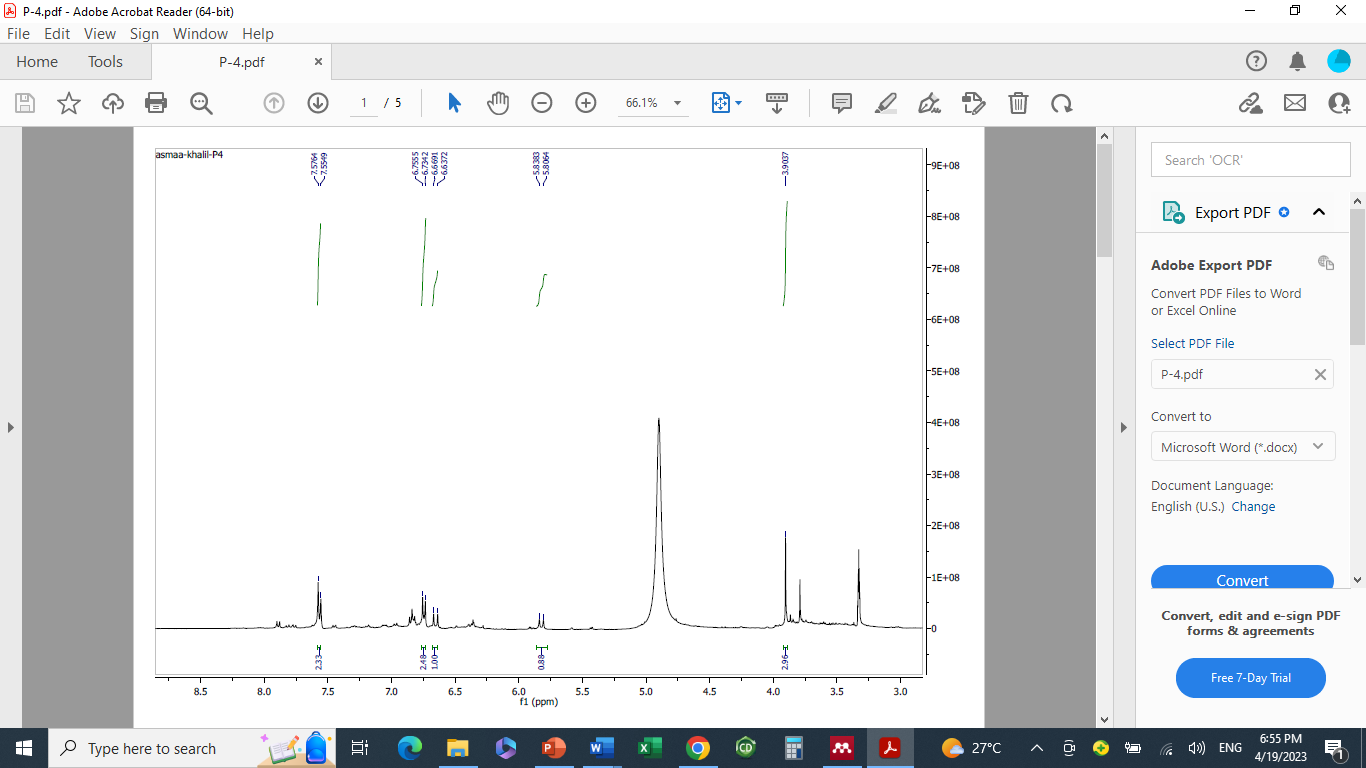


**Figure S31.** H^1^ NMR spectrum of methyl cis *p*-coumarate, component 3


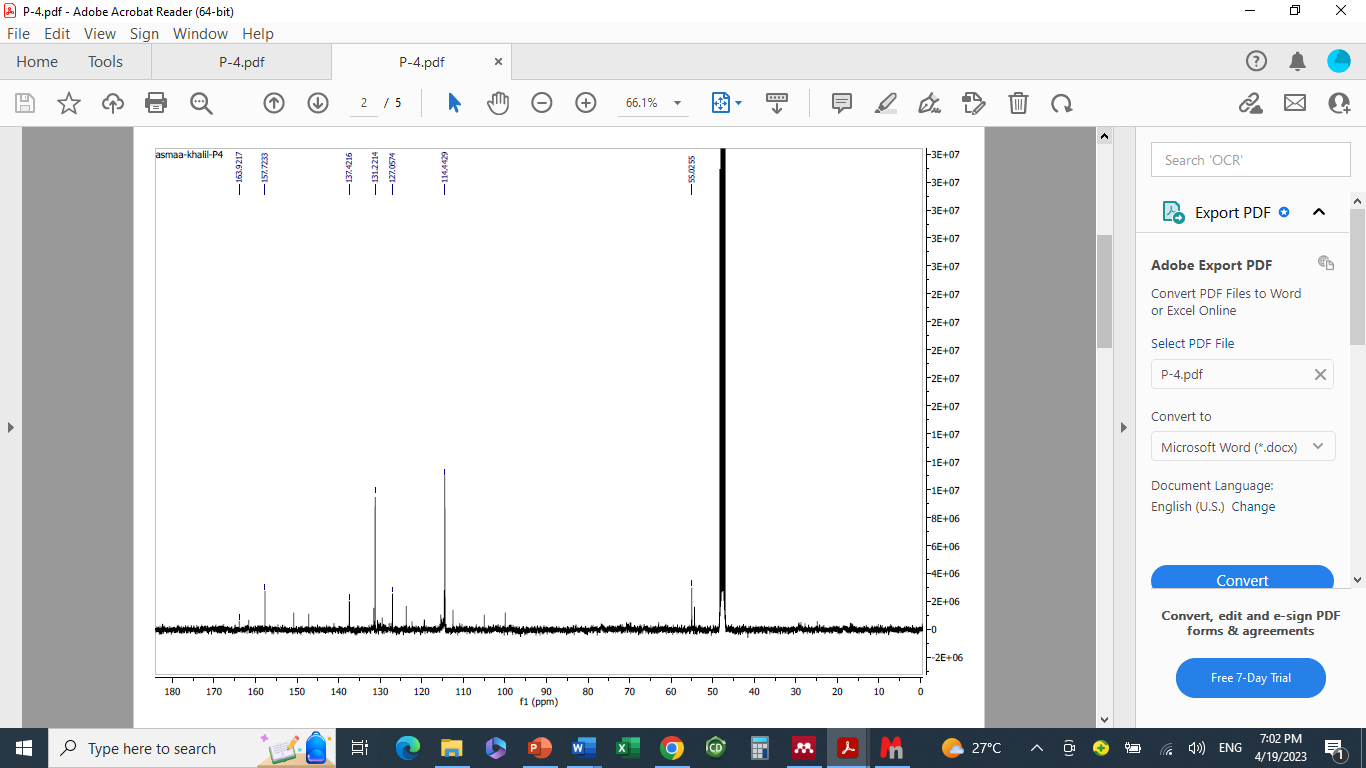


**Figure S32.** C^13^ NMR spectrum of methyl cis *p*-coumarate, component 3


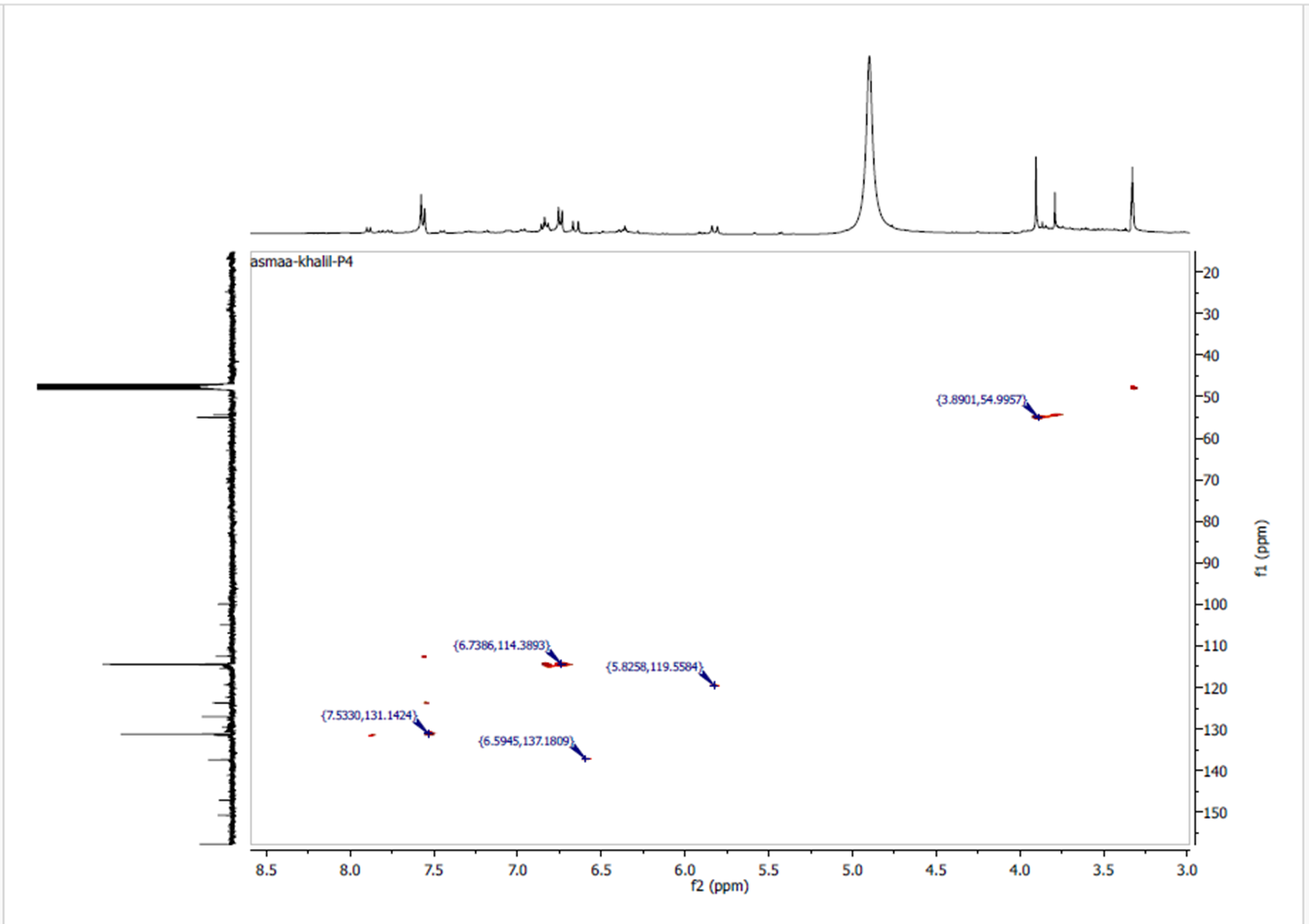


**Figure S33.** HSQC spectrum of methyl cis *p*-coumarate, component 3


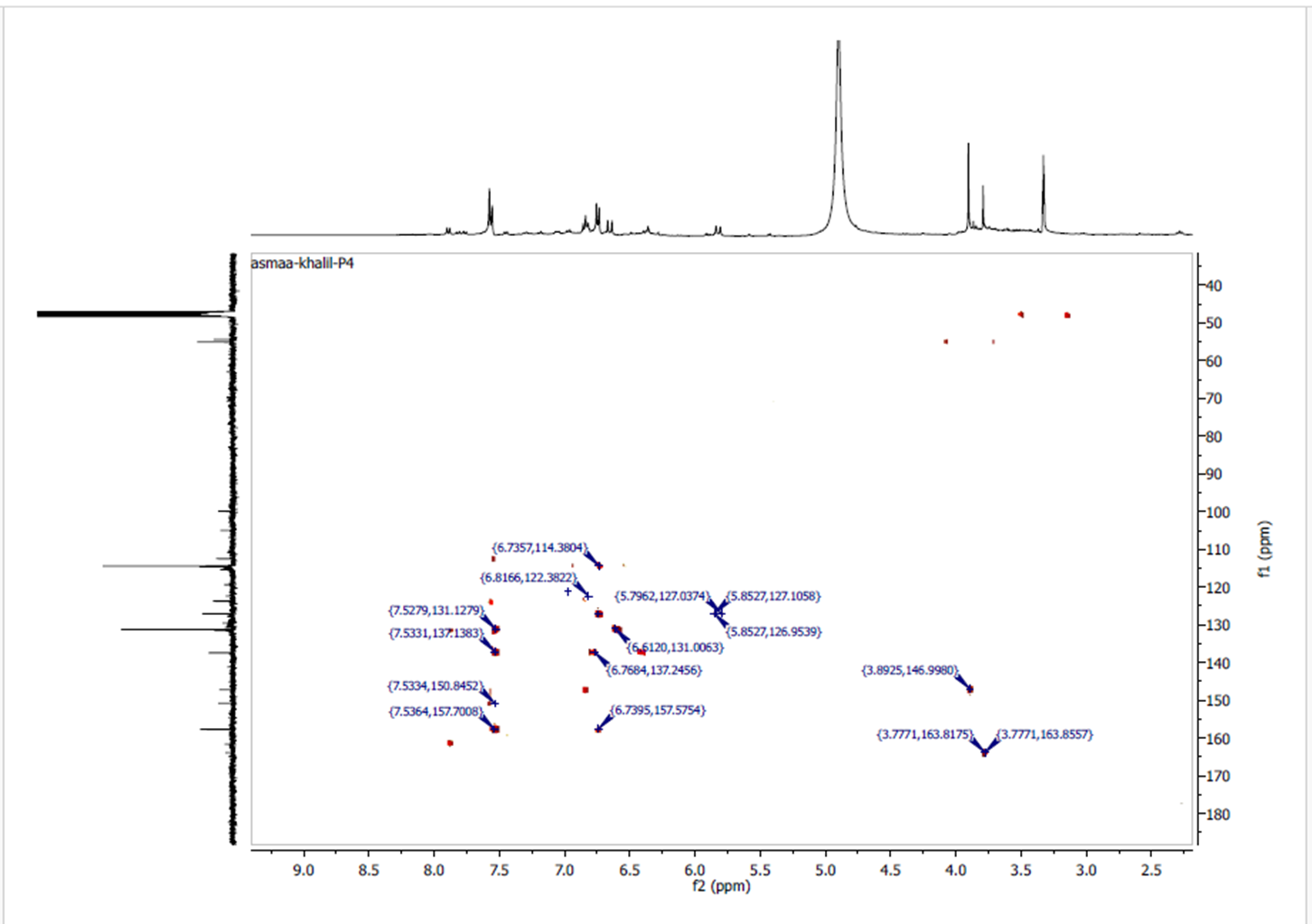


**Figure S34.** HMBC spectrum of methyl cis *p*-coumarate, component 3

**
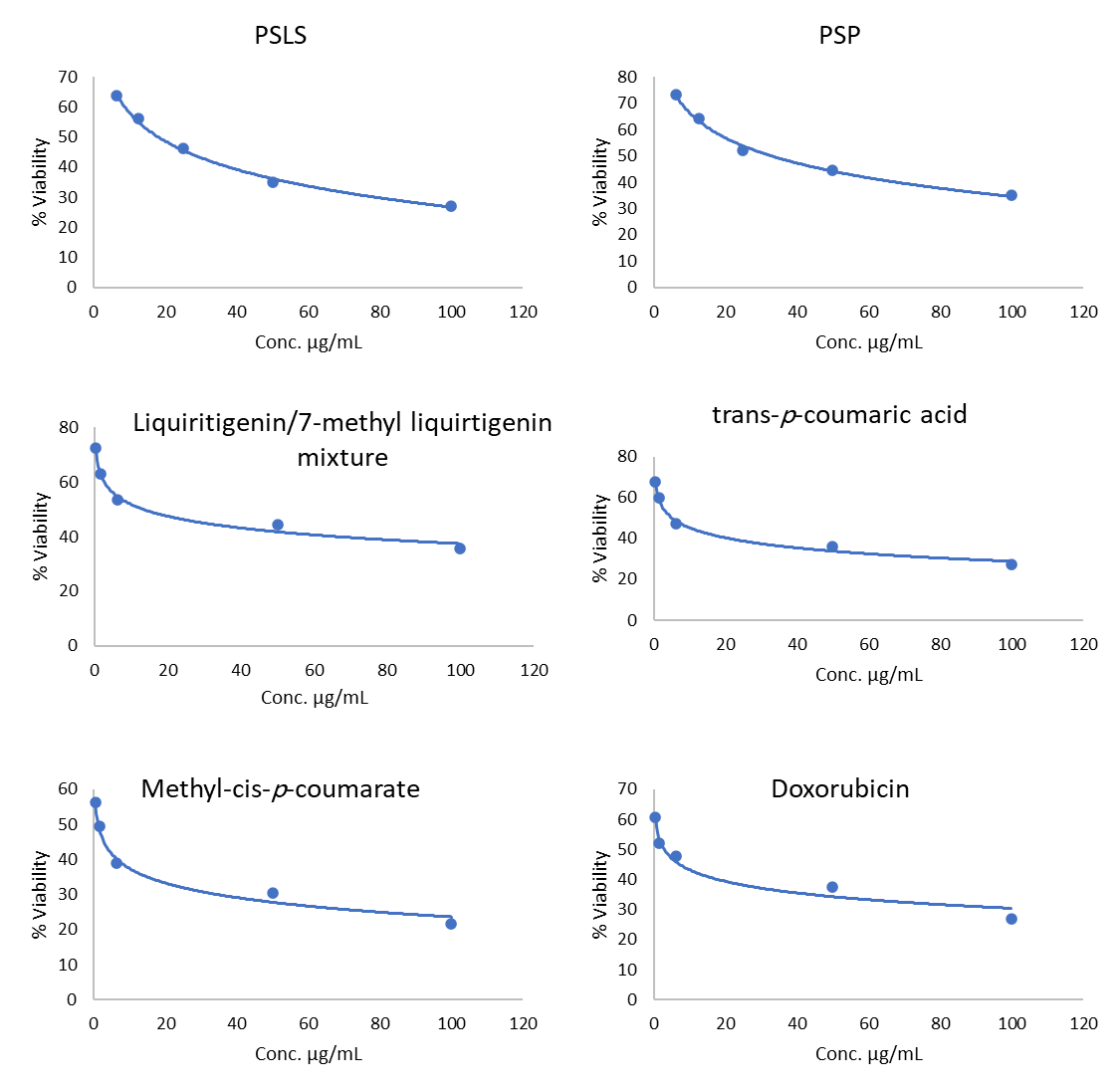
**

**Figure S35.** Percentage viability of MCF-7 cells at different concentrations of the investigated *Pisum sativum* L. (green pea, PS) waste extracts and isolated compounds from PSLS compared to doxorubicin.

PSLS: *Pisum sativum* leaves and stems extract. PSP: *Pisum sativum* peels extract.

**
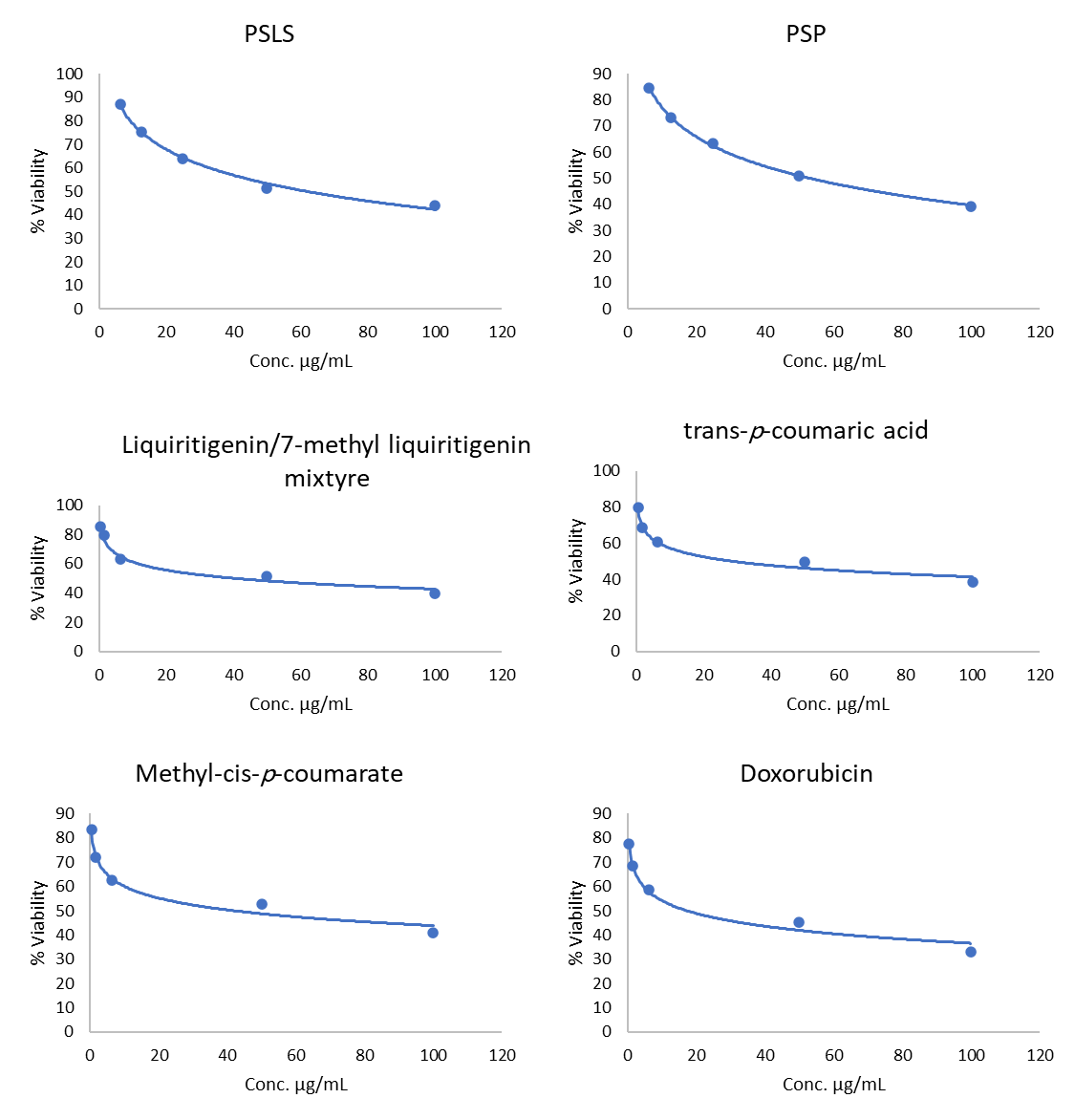
**

**Figure S36.** Percentage viability of MCF-10a cells with different concentrations of the investigated Pisum sativum L. (green pea, PS) waste extracts and isolated compounds compared to doxorubicin

PSLS: *Pisum sativum* leaves and stems extract. PSP: *Pisum sativum* peels extract.

**Table S1. Core targets of metabolites identified in *Pisum sativum* L. (green pea, PS) waste extracts by LC- MS/MS untargeted metabolomics, ranked according to the degree of involvement as revealed by network analysis.**

| **Target** | **Degree** | **Betweenness centrality** | **Closeness centrality** |
| --- | --- | --- | --- |
| CA12 (Carbonic anhydrase 12) | 64 | 0.003604 | 0.506316 |
| CA2 (Carbonic anhydrase 2) | 64 | 0.004186 | 0.506809 |
| CA1 (Carbonic anhydrase 1) | 62 | 0.002871 | 0.503381 |
| CYP19A1 (Cytochrome P19A1) | 56 | 0.002316 | 0.500480 |
| AKR1B1 (Aldo-keto reductase family 1 member B) | 56 | 0.002929 | 0.502410 |
| CA4 (Carbonic anhydrase 4) | 55 | 0.002584 | 0.501926 |
| CA9 (Carbonic anhydrase 9) | 52 | 0.002427 | 0.499521 |
| EGFR (Epidermal growth factor receptor) | 52 | 0.002108 | 0.498564 |
| ADORA3 (Adenosine A3 receptor) | 52 | 0.002541 | 0.498564 |
| PTPN1 (Protein tyrosine phosphatase non-receptor type 1) | 50 | 0.002529 | 0.491973 |
| ESR2 (Estrogen receptor 2) | 50 | 0.001534 | 0.490583 |
| ACHE (Acetylcholinesterase) | 50 | 0.001934 | 0.497612 |

**Table S2. The major hub targets ranked according to the degree of nodes sizes as revealed by PPI network analysis.**

| **Target** | **Degree** | **Betweenness centrality** | **Closeness centrality** |
| --- | --- | --- | --- |
| TP53 (Tumor protein p53) | 167 | 0.103117 | 0.474576 |
| AKT1 (AKT Serine/Threonine Kinase 1) | 122 | 0.053737 | 0.470971 |
| EGFR (Epidermal growth factor receptor) | 120 | 0.042200 | 0.458531 |
| SRC (Proto-oncogene tyrosine-protein kinase) | 114 | 0.041285 | 0.448115 |
| CTNNB1 (Catenin Beta 1) | 114 | 0.046893 | 0.457323 |
| HSP90AA1 (Heat shock protein 90kDa alpha, Class A, member 1) | 113 | 0.029875 | 0.445814 |
| STAT3 (Signal transducer and activator of transcription 3) | 107 | 0.022955 | 0.438827 |
| IL6 (Interleukin 6) | 99 | 0.027261 | 0.438827 |
| TNF (Tumor necrosis factor) | 88 | 0.024660 | 0.435524 |
| MAPK1 (Mitogen-activated protein kinase 1) | 88 | 0.021572 | 0.437941 |
| BCL2 (B-cell leukemia/lymphoma 2 protein) | 87 | 0.020397 | 0.434217 |
| MAPK3 (Mitogen-activated protein kinase 3) | 87 | 0.021139 | 0.433566 |
| ESR1 (Estrogen Receptor 1) | 84 | 0.030476 | 0.439939 |
| HSP90AB1 (Heat shock protein 90kDa alpha, Class B, member 1) | 83 | 0.012268 | 0.423414 |
| IL1B (Interleukin 1B) | 83 | 0.022993 | 0.431840 |
